# An application of MeSH enrichment analysis in livestock

**DOI:** 10.1111/age.12307

**Published:** 2015-06-02

**Authors:** G. Morota, F. Peñagaricano, J. L. Petersen, D. C. Ciobanu, K. Tsuyuzaki, I. Nikaido

**Affiliations:** ^1^ Department of Animal ScienceUniversity of NebraskaLincolnNEUSA; ^2^ Department of Animal SciencesUniversity of FloridaGainesvilleFLUSA; ^3^ University of Florida Genetics InstituteUniversity of FloridaGainesvilleFLUSA; ^4^ Department of Medicinal and Life ScienceFaculty of Pharmaceutical SciencesTokyo University of Science2641 YamazakiNodaChibaJapan; ^5^ Bioinformatics Research UnitAdvanced Center for Computing and CommunicationRIKEN2‐1 HirosawaWakoSaitamaJapan

**Keywords:** annotation, enrichment analysis, Gene Ontology, Gene, ORA

## Abstract

An integral part of functional genomics studies is to assess the enrichment of specific biological terms in lists of genes found to be playing an important role in biological phenomena. Contrasting the observed frequency of annotated terms with those of the background is at the core of overrepresentation analysis (ORA). Gene Ontology (GO) is a means to consistently classify and annotate gene products and has become a mainstay in ORA. Alternatively, Medical Subject Headings (MeSH) offers a comprehensive life science vocabulary including additional categories that are not covered by GO. Although MeSH is applied predominantly in human and model organism research, its full potential in livestock genetics is yet to be explored. In this study, MeSH ORA was evaluated to discern biological properties of identified genes and contrast them with the results obtained from GO enrichment analysis. Three published datasets were employed for this purpose, representing a gene expression study in dairy cattle, the use of SNPs for genome‐wide prediction in swine and the identification of genomic regions targeted by selection in horses. We found that several overrepresented MeSH annotations linked to these gene sets share similar concepts with those of GO terms. Moreover, MeSH yielded unique annotations, which are not directly provided by GO terms, suggesting that MeSH has the potential to refine and enrich the representation of biological knowledge. We demonstrated that MeSH can be regarded as another choice of annotation to draw biological inferences from genes identified via experimental analyses. When used in combination with GO terms, our results indicate that MeSH can enhance our functional interpretations for specific biological conditions or the genetic basis of complex traits in livestock species.

## Introduction

Identifying genes and genetic variants involved in the etiology of quantitative traits or complex diseases has received much attention in a wide spectrum of agricultural species (e.g., Goddard & Hayes 2009). This has been carried out via comparing changes in gene expression of thousands of loci derived from tissues in case–control studies. Alternatively, one can conduct large‐scale genome‐wide association studies or signatures of selection analyses using high‐density panels of genetic polymorphisms (e.g., SNPs). Regardless of the analysis methods employed, these studies generate lists of genes suggested to play a role in the variation of targeted traits. Typically, these lists are accompanied by mining biological function or biochemical pathways associated with a set of selected genes, which may aid in the dissection of the genetic basis of complex traits. Such analysis is often referred to as an overrepresentation analysis (ORA) by which one assesses whether any of annotations appear more frequently in a selected gene group relative to their occurrence by chance in a set of reference, background genes. This approach has been extensively used to help interpret the underlying genetic basis using lists of genes from selected loci since Drăghici *et al*. (2003). In general, ORA is coupled with structured biological annotations. Notably, enrichment analysis based on Gene Ontology (GO) attempts to uncover biological function(s) that the set of identified genes share with each other (Ashburner *et al*. 2000). This ontology comprises three categories for the purpose of annotating the biological knowledge of genes: Biological Process (BP), Cellular Component and Molecular Function (MF). Rich literature exists on the development of efficient methods, and a number of easily accessible software tools are available in the field of GO ORA; see Khatri & Drăghici (2005) and Rivals *et al*. (2007) for a review in the context of genome‐wide expression analysis.

Recently, the Medical Subject Headings (MeSH) vocabulary (e.g., Coletti & Bleich 2001; Nelson *et al*. 2004) has been making inroads into ORA. MeSH is a collection of comprehensive life sciences vocabulary, containing more than 25 000 clinical and biological annotations. MeSH is organized in a hierarchical structure and commonly each publication is associated with 10–15 headings. These headings represent key topics discussed in the papers and function to index articles in the MEDLINE database. In contrast to GO, in which functional ontologies are mapped to genes, a unique feature of MeSH is that terms are assigned to literature. Each MeSH descriptor is clustered into 16 categories; some MeSH categories, such as Diseases, are not covered in GO, but other categories, such as Chemicals and Drugs, and Phenomena and Processes, share similar concepts with those of GO (Osborne *et al*. 2007). As MeSH terms are assigned to PubMed literature, an additional step is required to associate MeSH to genes. Several computational tools, such as Gendoo (Nakazato *et al*. 2009), GeneMesh (Jani *et al*. 2010) and MeSHOPs (Cheung *et al*. 2012), associate genes with MeSH to permit finding statistically overrepresented MeSH annotations. Nakazato *et al*. (2008) argue that the total number of genes assigned to MeSH in Entrez Gene is larger than the number of genes assigned to GO, concluding that MeSH is better annotated than GO is. These studies have collectively spurred interest in the use of MeSH for mining biological concepts.

Despite the potential value of MeSH as annotations for enrichment analysis, pioneering efforts in MeSH is to a large extent limited to human and model organisms. One particular reason is that there is no tool available to support MeSH‐based analysis in livestock species. Recently, Tsuyuzaki *et al*. (2015) released a suite of MeSH ORA software by relating selected lists of genes to MeSH categories for domestic animals such as cattle, pig and horse. These are open source bioconductor packages in the r statistical programming environment and work seamlessly with each other.

The objective of this study was to conduct the first application of MeSH enrichment analysis for different livestock species based on data generated by three different approaches to demonstrate that MeSH ORA can be viewed as a useful, complementary tool for drawing biological interpretation of genes associated with specific conditions or traits. In the present note, we first describe the three datasets and elaborate on the details of the MeSH ORA framework applied. Then, we illustrate the application of MeSH ORA on candidate genes and/or quantitative trait loci (QTL) regions predicted to have functional roles in economically valuable traits in different livestock species and compare ontologies obtained through GO analysis. The paper concludes with a discussion on the utility of MeSH for providing another picture of the biological and genetic properties of the traits under study.

## Materials and methods

We used three datasets from previously published studies for the purpose of demonstrating MeSH ORA in animals. Briefly, these three datasets provide (i) genes that showed differential expression in preimplantation embryos due to maternal methionine supplementation in dairy cattle (Peñagaricano *et al*. 2013), (ii) genes from major QTL associated with age at puberty in gilts (Tart *et al*. 2013) and (iii) genes under selection in the Quarter Horse (Petersen *et al*. 2013). MeSH ID to Entrez Gene ID mapping tables for the three species were obtained from the org.mesh.bta.db, org.mesh.ssc.db and org.mesh.eqc.db packages for bovine, swine and equine respectively (These packages were renamed mesh.bta.eg.db, mesh.ssc.eg.db and mesh.eqc.eg.db respectively starting from bioconductor 3.1.) (Tsuyuzaki *et al*. 2015). The statistical significance of enrichment was assessed by a hypergeometric test (Adams & Skopek 1987) in the meshr package (Tsuyuzaki *et al*. 2015) to compute the probability that a certain annotation appears more than just by chance in the set of selected genes compared to that of a gene list of reference. The *P‐*value of the hypergeometric test is given by

$$P=\sum_{i=k}^{\min\;(M,n)}\frac{\left(\begin{array}{c} M\\ i \end{array}\right)\left(\begin{array}{c} N-M\\ n-i \end{array}\right)}{\left(\begin{array}{c} N\\ n \end{array}\right)},$$

where *N* is the total number of genes that were analyzed in the study (background genes), *M* is the total number of selected or significant genes, *n* is the total number of genes in the MeSH term under study, *k* is the number of selected or significant genes that belong to the MeSH term under study, and $\left(\begin{array}{c} y\\ x \end{array}\right)=y!/x!\;(y-x)!$ is the binomial coefficient. Likewise, we performed GO enrichment analysis using the annotation mapping packages org.bt.eg.db, org.ss.eg.db and go.db along with the gostats package to assess the significance of enrichment in bovine and swine datasets. Only MeSH ORA was performed for the Quarter Horse, as an official bioconductor genome annotation package for horse is not available yet. Given the set of input genes, the ORA framework returns a significantly overrepresented list of annotation terms relative to those annotated with the overall collection of genes. These highlighted enriched annotations are biological features postulated to be shared by the input gene sets. Although *P*‐values can be adjusted for multiple testing by choosing Benjamini–Hochberg, *Q*‐value and local false discovery rate methods in meshr, we opted not to consider this in an attempt to show a comprehensive picture of comparisons between MeSH and GO. Discussion regarding sensitivity and specificity in ORA via multiple‐testing corrections is, overall, still elusive (Huang da *et al*. 2009). All packages used in this analysis are available in version 2.14 and subsequent releases of bioconductor (http://bioconductor.org/). The cattle and Quarter Horse data are available under the GEO accession number (GSE48147) and at the AnimalGenome.ORG Data Repository (www.animalgenome.org/repository/pub/UMN2012.1130/) respectively.

## Results

Estimates of total numbers of genes annotated with MeSH and GO, the number of selected genes used as inputs in ORA and the numbers of selected genes annotated with MeSH and GO for the three datasets are described in Table 1.

**Table 1:** Description of the three datasets used. Annotated genes and input genes represent all the genes in the entire genome and selected genes of each species that are annotated by MeSH (Medical Subject Headings) and GO (Gene Ontology)

Species	Annotated genes		Input genes		
	MeSH	GO	Total	MeSH	GO
Cattle	17 130	17 652	222	207	186
Swine	15 786	15 663	152	50	136
Horse	9043	15 025	113	67	101

### Dairy cattle

The central hypothesis tested in Peñagaricano *et al*. (2013) study using RNA‐seq was that maternal methionine supplementation around conception can produce epigenetic changes (e.g., DNA methylation) in the fetal genome leading to changes in gene expression in early embryos. A subset of enriched MeSH terms significant at 5% (*P *≤* *0.05) associated with genes in which expression was altered by maternal methionine supplementation in dairy cattle is presented in Table 2. The hypothesis was supported by the MeSH heading Transcription, Genetic (MeSH:D014158) in the Phenomena and Processes category, whereas similar concepts were not picked up by GO terms (Appendix S1). Interestingly, MeSH ORA analysis captured some significant terms, such as Homocystine (MeSH:D006711) and Omega‐N‐Methylarginine (MeSH:D019323), that are related to the process of DNA methylation. Additional terms unique to MeSH are related to reproduction: Pregnancy, Animal (MeSH:D011270) and Embryonic Development (MeSH:D047108). Several MeSH annotations enriched for the Diseases category have connection to embryo development such as Abortion, Veterinary (MeSH:D000034); Placenta Diseases (MeSH:D010922); Pregnancy Complications (MeSH:D011248); and Embryo Loss (MeSH:D020964), all of which yield novel biological insights that are otherwise undetectable if only GO was used (Appendix S1). It is worth noting that several of these significant MeSH terms are associated with the *fibroblast growth factor 2* gene (*FGF2*), which was previously associated with early embryonic mortality in dairy cattle (Khatib *et al*. 2008). Remarkably, there is evidence that shows *FGF2* can be subject to epigenetic modifications, which in turn alter its expression during development (Li *et al*. 2008). Therefore, our MeSH analysis uncovers a potential role of the *FGF2* gene in the phenomenon of fetal programming due to maternal nutrition. Also, Mastitis, Bovine (MeSH:D008414), which has an implication to the immune system, was detected. The Chemicals and Drugs category identified several MeSH terms related to immune system such as Receptors, Immunologic (MeSH:D011971); Receptors, Natural Killer Cell (MeSH:D055607); Interferon‐gamma (MeSH:D007371); Receptors, Cytokine (MeSH:D018121); Interleukins (MeSH:D007378); and Histocompatibility Antigens Class II (MeSH:D000949). These MeSH terms tally with significantly enriched GO terms including Defense Response (GO:0006952), Immune Response (GO:0006955) and Response to Cytokine (GO:0034097) in the BP domain and Cytokine Receptor Binding (GO:0005126) in the MF category (Appendix S1). As pointed out by Peñagaricano *et al*. (2013), the alteration of the expression of genes related to innate and adaptive immune responses could have long‐term implications for the fitness of the offspring, which warrants further research.

**Table 2:** Statistically significant MeSH (Medical Subject Headings) terms related to genes that showed differential expression in preimplantation embryos due to maternal methionine supplementation in dairy cattle

Category	MeSH ID	MeSH term	Gene ID
Phenomena and Processes	D014158	Transcription, genetic	28116,282291,503620,281877
	D014158	Transcription, genetic	281429,790164,784768
	D011270	Pregnancy, Animal	281161,503620,280873
	D047108	Embryonic development	538769,280955,282291,281161
Diseases	D000034	Abortion, veterinary	282470
	D010922	Placenta diseases	282470
	D011248	Pregnancy complications	280843
	D020964	Embryo loss	281161
	D008414	Mastitis, bovine	613869,790164,281877,282470
	D008414	Mastitis, bovine	286836,282344
Chemicals and Drugs	D011971	Receptors, immunologic	790164,282467,444877,282390
	D055607	Receptors, natural killer cell	444877,282390
	D007371	Interferon‐gamma	281161,281214,282470,514889
	D018121	Receptors, cytokine	514889,281214
	D007378	Interleukins	282390,514889
	D006711	Homocystine	281161
	D019323	Omega‐N‐Methylarginine	281161
	D000949	Histocompatibility antigens class II	539241

### Swine

Age at puberty is a trait associated with the largest heritability of all reproductive traits in swine. The heritability estimate in the population resource targeted by Tart *et al*. (2013) was 38%, whereas the phenotypic variation explained by the Porcine SNP60 BeadChip (*n *=* *56 424 SNPs, Illumina) was 26%. The hypothesis of the Tart *et al*. study was that major QTL regions influencing variation of age at puberty most likely would include genes involved in an array of functions associated with reproductive processes, sexual and social behavior, energy metabolism, feed intake, etc.

A subset of statistically enriched MeSH terms that deserves particular attention in the area of puberty and energy metabolism in swine is highlighted in Table 3. In the Chemicals and Drugs category, Fatty Acid Synthase, Type I (MeSH:D054890) and Fatty Acid Synthases (MeSH:D064429) agreed with regulation of the Unsaturated Fatty Acid Biosynthetic Process (GO:2001279) in BP. Fatty acids have a role in energy production by ATP synthesis and are important for energy storage. Tart *et al*. (2013) found that energy intake significantly impacts expression of age at puberty and reported that a reduction in energy intake by 20% delayed age at puberty by about 7 days. One of the genes associated with MeSH:D054890 is *fatty acid synthase* (*FASN*). Polymorphisms located in *FASN* were evaluated as candidates for QTL for growth in cattle (Rempel *et al*. 2012) and for QTL for capric fatty acid and polyunsaturated fatty acids in sheep milk (García‐Fernández *et al*. 2010), whereas expression level of *FASN* was correlated with intramuscular fat in cattle (Jeong *et al*. 2012). These polymorphisms could act as potential pleiotropic variants influencing expression of puberty or fertility as well. This hypothesis was tested by Elis *et al*. (2013), who evaluated the expression of *FASN* and other adipokine and lipid metabolism genes in adipose tissue from dairy cows with difference in fertility. Likewise, Glycogen (MeSH:D006003), Glucose‐6‐Phosphate (MeSH:D019298), and Glucose Transporter Type 3 (MeSH:D051274) support Positive Regulation of Glycogen Catabolic Process (GO:0045819) in BP (Appendix S2). Glycogen is a multibranched form of glucose storage and energy source in animals. Only GO analysis uncovered BP clearly related to processes associated with expression of estrus including sexual behavior such as Negative Regulation of Female Receptivity (GO:0007621), maternal behavior such as Maternal Aggressive Behavior (GO:0002125) or blood circulation such as Regulation of Systemic Arterial Blood Pressure by Vasopressin (GO:0001992). The gene responsible for these functions is *arginine vasopressin receptor 1A* (*AVPR1A*). Tart *et al*. (2013) uncovered polymorphisms in *AVPR1A* associated with age at puberty and reproductive longevity in sows. Only MeSH analysis was able to uncover *ST3 beta‐galactoside alpha‐2,3‐sialyltransferase 1* (*ST3GAL1*), a gene member of the sialyltransferases family (MeSH:D012799) known to be influenced by level of progesterone in cyclic and pregnant female cattle (Sherblom *et al*. 1985).

**Table 3:** Statistically significant MeSH (Medical Subject Headings) terms related to age at puberty in swine

Category	MeSH ID	MeSH term	Gene ID
Chemicals and Drugs	D054890	Fatty acid synthase, type I	397561
	D064429	Fatty acid synthases	397561
	D006003	Glycogen	494561
	D019298	Glucose‐6‐Phosphate	494561
	D051274	Glucose transporter type 3	494561
	D012799	Sialyltransferases	445537

### Quarter Horse

In the horse, modern breeds have been intensely selected for specific characteristics based upon their intended use and criteria set by each breed registry. Due to this breed structure, which also includes closed or nearly closed populations, Petersen *et al*. (2013) hypothesized that regions harboring loci critical to breed‐specific phenotypes could be identified within genomic signatures of selection. Statistically significant MeSH terms identified from lists of genes underlying genomic signatures of selection in the Quarter Horse are shown in Table 4. The Quarter Horse is known for its sprinting ability and heavy muscling; Petersen *et al*. (2013) has shown that variants in the *myostatin* gene (*MSTN*) are associated with skeletal muscle fiber type proportions more suited for sprinting. Fittingly, these findings are corroborated by the MeSH term Myostatin (MeSH:D055435) in the Chemicals and Drugs category. *MSTN* also underlies MeSH terms Physical Conditioning, Animal (MeSH:D010805), Somatotypes (MeSH:D013008) and Base Pairing (MeSH:D020029) in the category Phenomena and Processes (Appendix S3). Other MeSH terms include Gait (MeSH:D005684), Motor Activity (MeSH:D009043), Running (MeSH:D012420) and Physical Endurance (MeSH:D010807). The genes from which these MeSH terms were derived include *tumor necrosis factor* (*TNF*) and *double‐sex and mab‐related transcription factor 3* (*DMRT3*). *TNF* is a proinflammatory cytokine that has been suggested to activate NF‐κB signaling in skeletal muscle (Bhatnagar *et al*. 2010), whereas *DMRT3* is associated with the ability of some breeds of horse to perform alternative gaits. Although the presence of alternative gaits is deemed undesirable in the Quarter Horse, this locus may appear as the result of negative selection or due to the allelic frequency in the population with which the comparison was performed. Because these terms are not directly covered in GO, MeSH provides an easily comprehensible interpretation of the genes.

**Table 4:** Statistically significant MeSH (Medical Subject Headings) terms related to genes under selection in Quarter Horse

Category	MeSH ID	MeSH term	Gene ID
Chemicals and drugs	D055435	Myostatin	100033832
Phenomena and processes	D010805	Physical conditioning, animal	100033832, 100033834
	D013008	Somatotypes	100033832
	D020029	Base pairing	100033832
	D005684	Gait	100147177
	D009043	Motor activity	100033832
	D012420	Running	100033832
	D010807	Physical endurance	100033834

Source code and output reports generated by r markdown are available as pdfs in Appendices S1, S2 and S3. Taken together, our results indicate that MeSH ORA opens the possibility of discovery of relevant biological phenomena from genes of interest.

## Discussion

Our quest for searching for genes and genetic variants responsible for the variation of economically important traits in livestock species is supported in the post‐genomic era by the sheer quantity of genetic polymorphisms. Annotation of genes is an increasingly important challenge to fully dissect research findings from large‐scale gene expression or SNP‐based genome‐wide association analyses. Thus, the functional characterization of putatively selected loci becomes more and more crucial as we delve into deciphering the underlying genetic basis of multifactorial traits (Soldatos *et al*. 2015). Further, rich annotation offers a new avenue to investigate the source of predictive ability for each annotated genomic region via a genome partitioning approach (Morota *et al*. 2014). Although DNA‐based enrichment analysis needs to be carefully applied when moving from variant‐level to gene‐level inference (Mirina *et al*. 2012; Sedeno‐Cortés & Pavlidis 2014), the knowledge gained through GO and similar efforts to characterize genes should receive more consideration in livestock species. For instance, the lack of available software tools has posed a challenge in assessing the enrichment of specific MeSH annotations in livestock for decades. Thereby, through this study, we strived to demonstrate that MeSH enrichment analysis has a potential to aid in the functional interpretation of genes of interest. One potential caveat of ORA is how reliable the assignment of MeSH and GO terms to gene products is. For example, more than half of GO annotations are assigned to bovine genes with evidence codes of IEA (inferred from electronic annotation; http://geneontology.org/page/download-annotations). Similarly, because MeSH annotations for some minor organisms were assigned based on sequence similarity, a cautious interpretation of results is always advisable (Tsuyuzaki *et al*. 2015).

Given that MeSH enrichment statistics rely on a great body of scientific literature, reliability of outcomes should improve over time as new knowledge gets disseminated via peer‐reviewed articles. We expect that genes will become better annotated using MeSH with the increase in the number of high‐quality publications. Notably, the demands for data interpretation are more pressing than ever before due to the massive amount of data being generated. MeSH ORA is now available for livestock species as well to meet this new challenge. When taken together with GO analysis, MeSH enrichment analysis may help to provide additional insight into biological interpretation of genes influencing complex traits.

## Conclusions

We deployed the National Library of Medicine's controlled MeSH vocabulary, which has gained less attention in non‐model organisms such as livestock species, for high‐throughput data interpretation via literature‐derived concepts. The main goal of this study was to identify statistically significant enriched annotations among a given list of genes. We found that some overrepresented MeSH terms linked to selected genes share similar concepts derived from GO annotations in three agricultural species: dairy cattle, swine and Quarter Horse. Moreover, we were able to draw additional information not directly provided by GO. This implies MeSH enrichment analysis can be regarded as a supplementary tool to GO analysis. Although inferring the molecular mechanism on the basis of statistically significant MeSH or GO annotations alone is indeed potentially error‐prone (Pavlidis *et al*. 2012), we contend that ORA is still a critical first step toward inferring biological interpretations of gene sets potentially associated with important genetic roles in the phenotypic variation.

## Competing interests

The authors declare that they have no competing interests.

## Authors' contributions

GM, KT and IN conceived the study. FP, JLP and DCC prepared the data. GM analyzed the data and drafted the manuscript. GM, FP, JLP, DCC, KT and IN revised the manuscript. All authors read and approved the final manuscript.

## Supporting information


**Appendix S1** MeSH over‐representation analysis (Bovine)

**Appendix S2** MeSH over‐representation analysis (Swine)

**Appendix S3** MeSH over‐representation analysis (Horse)

## References

[age12307-bib-0001] Adams W.T. & Skopek T.R. (1987) Statistical test for the comparison of samples from mutational spectra. Journal of Molecular Biology 194, 391–6. 3305960 10.1016/0022-2836(87)90669-3

[age12307-bib-0002] Ashburner M. , Ball C.A. , Blake J.A. , *et al* (2000) Gene Ontology: tool for the unification of biology. Nature Genetics 25, 25–9. 10802651 10.1038/75556 PMC3037419

[age12307-bib-0003] Bhatnagar S. , Panguluri S.K. , Gupta S.K. , Dahiya S. , Lundy R.F. & Kumar A. (2010) *Tumor necrosis factor‐*α regulates distinct molecular pathways and gene networks in cultured skeletal muscle cells. PLoS ONE 5, e13262. 20967264 10.1371/journal.pone.0013262 PMC2953497

[age12307-bib-0004] Cheung W.A. , Francis Ouellette B.F. & Wasserman W.W. (2012) Quantitative biomedical annotation using medical subject heading over‐representation profiles (MeSHOPs). BMC Bioinformatics 13, 249. 23017167 10.1186/1471-2105-13-249 PMC3564935

[age12307-bib-0005] Coletti M.H. & Bleich H.L. (2001) Medical subject headings used to search the biomedical literature. Journal of the American Medical Informatics Association 8, 317–23. 11418538 10.1136/jamia.2001.0080317 PMC130076

[age12307-bib-0006] Drăghici S. , Khatria P. , Martinsb R.P. , Ostermeier G.C. & Krawetz S.A. (2003) Global functional profiling of gene expression. Genomics 81, 98–104. 12620386 10.1016/s0888-7543(02)00021-6

[age12307-bib-0007] Elis S. , Coyral‐Castel S. , Freret S. , Cognié J. , Desmarchais A. , Fatet A. , Rame C. , Briant E. , Maillard V. & Dupont J. (2013) Expression of adipokine and lipid metabolism genes in adipose tissue of dairy cows differing in a female fertility quantitative trait locus. Journal of Dairy Science 96, 7591–602. 24119802 10.3168/jds.2013-6615

[age12307-bib-0008] García‐Fernández M. , Gutiérrez‐Gil B. , García‐Gámez E. , Sánchez J.P. & Arranz J.J. (2010) The identification of QTL that affect the fatty acid composition of milk on sheep chromosome 11. Animal Genetics 41, 324–8. 19968648 10.1111/j.1365-2052.2009.02000.x

[age12307-bib-0009] Goddard M.E. & Hayes B.J. (2009) Mapping genes for complex traits in domestic animals and their use in breeding programmes. Nature Reviews Genetics 10, 381–91. 10.1038/nrg2575 19448663

[age12307-bib-0010] Huang da W. , Sherman B.T. & Lempicki R.A. (2009) Bioinformatics enrichment tools: paths toward the comprehensive functional analysis of large gene lists. Nucleic Acids Research 37, 1–13. 19033363 10.1093/nar/gkn923 PMC2615629

[age12307-bib-0011] Jani S.D. , Argraves G.L. , Barth J.L. & Argraves W.S. (2010) genemesh: a web‐based microarray analysis tool for relating differentially expressed genes to MeSH terms. BMC Bioinformatics 11, 166. 20359363 10.1186/1471-2105-11-166 PMC3212930

[age12307-bib-0012] Jeong J. , Kwon E.G. , Im S.K. , Seo K.S. & Baik M. (2012) Expression of fat deposition and fat removal genes is associated with intramuscular fat content in longissimus dorsi muscle of Korean cattle steers. Journal of Animal Science 90, 2044–53. 22266990 10.2527/jas.2011-4753

[age12307-bib-0013] Khatib H. , Maltecca C. , Monson R.L. , Schutzkus V. , Wang X. & Rutledge J.J. (2008) The *fibroblast growth factor 2* gene is associated with embryonic mortality in cattle. Journal of Animal Science 86, 2063–7. 18469054 10.2527/jas.2007-0791

[age12307-bib-0014] Khatri P. & Drăghici S. (2005) Ontological analysis of gene expression data: current tools, limitations, and open problems. Bioinformatics 21, 3587–95. 15994189 10.1093/bioinformatics/bti565 PMC2435250

[age12307-bib-0015] Li X. , Barkho B.Z. , Luo Y. , Smrt R.D. , Santistevan N.J. , Liu C. , Kuwabara T. , Gage F.H. & Zhao X. (2008) Epigenetic regulation of the stem cell mitogen Fgf‐2 by Mbd1 in adult neural stem/progenitor cells. The Journal of Biological Chemistry 283, 27644–52. 18689796 10.1074/jbc.M804899200 PMC2562066

[age12307-bib-0016] Mirina A. , Atzmon G. , Ye K. & Bergman A. (2012) Gene size matters. PLoS ONE 7, e49093. 23152854 10.1371/journal.pone.0049093 PMC3494661

[age12307-bib-0017] Morota G. , Abdollahi‐Arpanahi R. , Kranis A. & Gianola D. (2014) Genome‐enabled prediction of quantitative traits in chickens using genomic annotation. BMC Genomics 15, 109. 24502227 10.1186/1471-2164-15-109 PMC3922252

[age12307-bib-0018] Nakazato T. , Bono H. , Matsuda H. & Takagi T. (2009) Gendoo: functional profiling of gene and disease features using MeSH vocabulary. Nucleic Acids Research 37, W166–9. 19498079 10.1093/nar/gkp483 PMC2703956

[age12307-bib-0019] Nakazato T. , Takinaka T. , Mizuguchi H. , Matsuda H. , Bono H. & Asogawa M. (2008) BioCompass: a novel functional inference tool that utilizes MeSH hierarchy to analyze groups of genes. In Silico Biology 8, 53–61. 18430990

[age12307-bib-0020] Nelson S.J. , Schopen M. , Savage A.G. , Schulman J.L. & Arluk N. (2004) The MeSH translation maintenance system: structure, interface design, and implementation. Studies in Health Technology and Informatics 107, 67–9. 15360776

[age12307-bib-0021] Osborne J.D. , Zhu L.J. , Lin S.M. & Kibbe W.A. (2007) Interpreting microarray results with Gene Ontology and MeSH. Methods in Molecular Biology 29, 223–41. 17634620 10.1007/978-1-59745-390-5_14

[age12307-bib-0022] Pavlidis P. , Jensen J.D. , Stephan W. & Stamatakis A. (2012) A critical assessment of storytelling: Gene Ontology categories and the importance of validating genomic scans. Molecular Biology and Evolution 29, 3237–48. 22617950 10.1093/molbev/mss136

[age12307-bib-0023] Peñagaricano F. , Souza A.H. , Carvalho P.D. , Driver A.M. , Gambra R. , Kropp J. , Hackbart K.S. *et al* (2013) Effect of maternal methionine supplementation on the transcriptome of bovine preimplantation embryos. PLoS ONE 8, e72302. 23991086 10.1371/journal.pone.0072302 PMC3749122

[age12307-bib-0024] Petersen J.L. , Mickelson J.R. , Rendahl A.K. , *et al* (2013) Genome‐wide analysis reveals selection for important traits in domestic horse breeds. PLOS Genetics 9, e1003211. 23349635 10.1371/journal.pgen.1003211 PMC3547851

[age12307-bib-0025] Petersen J.L. , Valberg S.J. , Mickelson J.R. & McCue M.E. (2014) Haplotype diversity in the equine *myostatin* gene with focus on variants associated with race distance propensity and muscle fiber type proportions. Animal Genetics 45, 827–35. 25160752 10.1111/age.12205 PMC4211974

[age12307-bib-0026] Rempel L.A. , Casas E. , Shackelford S.D. & Wheeler T.L. (2012) Relationship of polymorphisms within metabolic genes and carcass traits in crossbred beef cattle. Journal of Animal Science 90, 1311–6. 22100592 10.2527/jas.2011-4302

[age12307-bib-0027] Rivals I. , Personnaz L. , Taing L. & Potier M.C. (2007) Enrichment or depletion of a GO category within a class of genes: which test? Bioinformatics 23, 401–7. 17182697 10.1093/bioinformatics/btl633

[age12307-bib-0028] Sedeno‐Cortés A.E. & Pavlidis P. (2014) Pitfalls in the application of gene‐set analysis to genetics studies. Trends in Genetics 30, 513–4. 25459301 10.1016/j.tig.2014.10.001 PMC5369409

[age12307-bib-0029] Sherblom A.P. , Smagula R.M. , Moody C.E. & Anderson G.W. (1985) Immunosuppression, sialic acid, and sialyltransferase of bovine serum as a function of progesterone concentration. Journal of Reproduction and Fertility 74, 509–17. 4045819 10.1530/jrf.0.0740509

[age12307-bib-0030] Soldatos T.G. , Perdigão N. , Brown N.P. , Sabir K.S. & O'Donoghue S.I. (2015) How to learn about gene function: text‐mining or ontologies? Methods 74, 3–15. 25088781 10.1016/j.ymeth.2014.07.004

[age12307-bib-0031] Tart J.K. , Johnson R.K. , Bundy J.W. , *et al* (2013) Genome‐wide prediction of age at puberty and reproductive longevity in sows. Animal Genetics 44, 387–97. 23437861 10.1111/age.12028

[age12307-bib-0032] Tsuyuzaki K. , Morota G. , Ishii M. , Nakazato T. , Miyazaki S. & Nikaido I. (2015) MeSH ORA framework: r/bioconductor packages to support MeSH over‐representation analysis. BMC Bioinformatics 16, 45. 25887539 10.1186/s12859-015-0453-z PMC4343279

