# Supplementary material for: An application of MeSH enrichment analysis in livestock
Source: Anim Genet. 2015 Jun 2;46(4):381–7. doi: 10.1111/age.12307 (PMC5032990; doi:10.1111/age.12307)
Supplement: Supplementary file 1 — Appendix S1 MeSH over‐representation analysis (Bovine) [file AGE-46-381-s001.pdf]

APPENDIX S1

MeSH over-representation analysis (Bovine)

This is a reproducible report written by an R Markdown.

1. Create a vector of all genes

We first create a vector of all genes.

```
library("org.Bt.eg.db")
key.symbol <- keys(org.Bt.eg.db,  keytype = c("SYMBOL"))
entrezUniverse = select(org.Bt.eg.db, as.character(key.symbol), columns= c("ENTREZID", "ENSEMBL"),
                        keytype = "SYMBOL")  # 45937
```

```
## Warning in .generateExtraRows(tab, keys, jointype): 'select' resulted in
## 1:many mapping between keys and return rows
```

```
## remove duplicated Entrez Gene ID
entrezUniverse2 <- entrezUniverse[!duplicated(entrezUniverse[,2]),] # 44955
## remove duplicated Gene Symbol
entrezUniverse3 <- entrezUniverse2[!duplicated(entrezUniverse2[,1]),] # 36634
```

2. Create a vector of background (universe) genes

Then we make a vector of background (universe) genes. This object will be used throughout the following analysis. We read the file *list.full.genes.txt*.

```
genes.univ.cattle.org <- read.table("list.full.genes.txt", colClasses = c("character"), header=FALSE)
colnames(genes.univ.cattle.org) <- "SYMBOL"
## merge two files
my.univ.geneID.org <- merge(genes.univ.cattle.org, entrezUniverse3, by ="SYMBOL") # 9434
## remove duplicated Entrez Gene ID
my.univ.geneID2.org <- my.univ.geneID.org[ !duplicated(my.univ.geneID.org[,2]),] # 9372
```

3. Create a vector of significant genes

Thirdly, we create a vector of significant genes by reading the file *list.sig.genes.txt*.

```
## read data
genes.cattle.org <- read.table("list.sig.genes.txt", colClasses = c(rep("NULL", 4), "character"),
                               header=TRUE)
colnames(genes.cattle.org) <- "SYMBOL"
## merge two files
my.geneID.org <- merge(genes.cattle.org, entrezUniverse3, by ="SYMBOL") # 222
## remove duplicated Entrez Gene ID
my.geneID2.org <- my.geneID.org[ !duplicated(my.geneID.org[,2]),] # 222
```

4. GO enrichment analysis

We perform a GO analysis via the *GOstats* package.

```
library("GOstats")
paraGO <- new("GOHyperGParams", geneIds=my.geneID2.org[,2], universeGeneIds=my.univ.geneID2.org[,2],
              annotation="org.Bt.eg.db", ontology="BP",
              pvalueCutoff=0.05, conditional=TRUE, testDirection="over")
```

GO enrichment analysis for **BP**

```
BP <- hyperGTest(paraGO)
summary(BP)[,c(1,2,7)]
```

```
##        GOBPID       Pvalue
## 1  GO:0006954 0.0006079206
## 2  GO:0070098 0.0009115902
## 3  GO:0006955 0.0023174504
## 4  GO:0030574 0.0029700738
## 5  GO:0007601 0.0029881189
## 6  GO:0098656 0.0035411419
## 7  GO:0010001 0.0048249955
## 8  GO:0070588 0.0048249955
## 9  GO:0007186 0.0049613925
## 10 GO:1902476 0.0060967645
## 11 GO:0098660 0.0069780201
## 12 GO:0045595 0.0071961809
## 13 GO:0023052 0.0078972982
## 14 GO:0042330 0.0082419568
## 15 GO:0007417 0.0146624328
## 16 GO:0015698 0.0152595369
## 17 GO:0097192 0.0152595369
## 18 GO:0097529 0.0152595369
## 19 GO:0045087 0.0153227962
## 20 GO:0000189 0.0176933159
## 21 GO:0006700 0.0176933159
## 22 GO:0010744 0.0176933159
## 23 GO:0010807 0.0176933159
## 24 GO:0010886 0.0176933159
## 25 GO:0014002 0.0176933159
## 26 GO:0015820 0.0176933159
## 27 GO:0030644 0.0176933159
## 28 GO:0034769 0.0176933159
## 29 GO:0035494 0.0176933159
## 30 GO:0048714 0.0176933159
## 31 GO:0051902 0.0176933159
## 32 GO:0055083 0.0176933159
## 33 GO:2000110 0.0176933159
## 34 GO:2001023 0.0176933159
## 35 GO:2001223 0.0176933159
## 36 GO:0044259 0.0181055841
## 37 GO:0050920 0.0181055841
## 38 GO:0007165 0.0191140673
```

```
## 39 GO:0007229 0.0211572548
## 40 GO:0048592 0.0211572548
## 41 GO:0007600 0.0229881550
## 42 GO:0070838 0.0247698094
## 43 GO:0030595 0.0278461291
## 44 GO:0050767 0.0305517576
## 45 GO:0045321 0.0309204946
## 46 GO:0017157 0.0314681736
## 47 GO:0098655 0.0316558169
## 48 GO:0042119 0.0344577367
## 49 GO:0002275 0.0344689408
## 50 GO:0002281 0.0350792749
## 51 GO:0002283 0.0350792749
## 52 GO:0002504 0.0350792749
## 53 GO:0002540 0.0350792749
## 54 GO:0008045 0.0350792749
## 55 GO:0017186 0.0350792749
## 56 GO:0030002 0.0350792749
## 57 GO:0033032 0.0350792749
## 58 GO:0034367 0.0350792749
## 59 GO:0034369 0.0350792749
## 60 GO:0034372 0.0350792749
## 61 GO:0035524 0.0350792749
## 62 GO:0035845 0.0350792749
## 63 GO:0043217 0.0350792749
## 64 GO:0043303 0.0350792749
## 65 GO:0045494 0.0350792749
## 66 GO:0045576 0.0350792749
## 67 GO:0046548 0.0350792749
## 68 GO:0046668 0.0350792749
## 69 GO:0060020 0.0350792749
## 70 GO:0071825 0.0350792749
## 71 GO:0090077 0.0350792749
## 72 GO:1901741 0.0350792749
## 73 GO:2001204 0.0350792749
## 74 GO:0070887 0.0356127659
## 75 GO:0071345 0.0392729270
## 76 GO:0007423 0.0416316214
## 77 GO:0007267 0.0421664725
## 78 GO:0006950 0.0429577805
## 79 GO:0002263 0.0433579109
## 80 GO:0042742 0.0433579109
## 81 GO:0048754 0.0433579109
##                                                                Term
## 1                                              inflammatory response
## 2                                   chemokine-mediated signaling pathway
## 3                                                    immune response
## 4                                          collagen catabolic process
## 5                                                  visual perception
## 6                                       anion transmembrane transport
## 7                                         glial cell differentiation
## 8                                  calcium ion transmembrane transport
## 9                           G-protein coupled receptor signaling pathway
## 10                                   chloride transmembrane transport
```

```
## 11                                                   inorganic ion transmembrane transport
## 12                                                     regulation of cell differentiation
## 13                                                                              signaling
## 14                                                                                  taxis
## 15                                                     central nervous system development
## 16                                                               inorganic anion transport
## 17                                   extrinsic apoptotic signaling pathway in absence of ligand
## 18                                                            myeloid leukocyte migration
## 19                                                                 innate immune response
## 20                                                                MAPK import into nucleus
## 21                                                    C21-steroid hormone biosynthetic process
## 22                          positive regulation of macrophage derived foam cell differentiation
## 23                                                      regulation of synaptic vesicle priming
## 24                                                  positive regulation of cholesterol storage
## 25                                                                   astrocyte development
## 26                                                                       leucine transport
## 27                                                         cellular chloride ion homeostasis
## 28                                                            basement membrane disassembly
## 29                                                                SNARE complex disassembly
## 30                                     positive regulation of oligodendrocyte differentiation
## 31                                       negative regulation of mitochondrial depolarization
## 32                                                    monovalent inorganic anion homeostasis
## 33                                        negative regulation of macrophage apoptotic process
## 34                                                             regulation of response to drug
## 35                                                     negative regulation of neuron migration
## 36                                     multicellular organismal macromolecule metabolic process
## 37                                                                  regulation of chemotaxis
## 38                                                                       signal transduction
## 39                                                         integrin-mediated signaling pathway
## 40                                                                       eye morphogenesis
## 41                                                                       sensory perception
## 42                                                               divalent metal ion transport
## 43                                                                     leukocyte chemotaxis
## 44                                                                 regulation of neurogenesis
## 45                                                                     leukocyte activation
## 46                                                                  regulation of exocytosis
## 47                                                             cation transmembrane transport
## 48                                                                      neutrophil activation
## 49                                       myeloid cell activation involved in immune response
## 50                                         macrophage activation involved in immune response
## 51                                         neutrophil activation involved in immune response
## 52  antigen processing and presentation of peptide or polysaccharide antigen via MHC class II
## 53                                     leukotriene production involved in inflammatory response
## 54                                                                 motor neuron axon guidance
## 55 peptidyl-pyroglutamic acid biosynthetic process, using glutaminyl-peptide cyclotransferase
## 56                                                                 cellular anion homeostasis
## 57                                              regulation of myeloid cell apoptotic process
## 58                                                          macromolecular complex remodeling
## 59                                                     plasma lipoprotein particle remodeling
## 60                                           very-low-density lipoprotein particle remodeling
## 61                                                              proline transmembrane transport
## 62                                             photoreceptor cell outer segment organization
## 63                                                                        myelin maintenance
## 64                                                                    mast cell degranulation
```

```
## 65                                   photoreceptor cell maintenance
## 66                                          mast cell activation
## 67                                 retinal rod cell development
## 68              regulation of retinal cell programmed cell death
## 69                        Bergmann glial cell differentiation
## 70                   protein-lipid complex subunit organization
## 71                                    foam cell differentiation
## 72                       positive regulation of myoblast fusion
## 73                         regulation of osteoclast development
## 74                        cellular response to chemical stimulus
## 75                        cellular response to cytokine stimulus
## 76                                    sensory organ development
## 77                                          cell-cell signaling
## 78                                           response to stress
## 79                 cell activation involved in immune response
## 80                                 defense response to bacterium
## 81                  branching morphogenesis of an epithelial tube
```

GO enrichment analysis for **MF**

```
ontology(paraGO) <- "MF"
MF <- hyperGTest(paraGO)
summary(MF)[,c(1,2,7)]
```

```
##        GOMFID       Pvalue
## 1  GO:0005215 0.0002468818
## 2  GO:0001968 0.0008930110
## 3  GO:0008009 0.0008930110
## 4  GO:0043394 0.0008930110
## 5  GO:0005262 0.0012789322
## 6  GO:0008201 0.0040241894
## 7  GO:0072509 0.0040241894
## 8  GO:0005216 0.0043609993
## 9  GO:0015267 0.0052333776
## 10 GO:0046873 0.0052333776
## 11 GO:0022832 0.0053891818
## 12 GO:0022891 0.0075549647
## 13 GO:0005254 0.0078795607
## 14 GO:0005518 0.0078795607
## 15 GO:0008324 0.0079429281
## 16 GO:0001948 0.0092431400
## 17 GO:0015103 0.0149672001
## 18 GO:0022843 0.0149672001
## 19 GO:0001637 0.0175310446
## 20 GO:0004383 0.0175310446
## 21 GO:0004465 0.0175310446
## 22 GO:0004613 0.0175310446
## 23 GO:0005298 0.0175310446
## 24 GO:0005416 0.0175310446
## 25 GO:0008331 0.0175310446
## 26 GO:0008445 0.0175310446
## 27 GO:0016494 0.0175310446
## 28 GO:0019905 0.0175310446
```

```
## 29 GO:0031433 0.0175310446
## 30 GO:0031726 0.0175310446
## 31 GO:0032027 0.0175310446
## 32 GO:0034185 0.0175310446
## 33 GO:0005126 0.0333439962
## 34 GO:0003796 0.0347610445
## 35 GO:0004090 0.0347610445
## 36 GO:0004890 0.0347610445
## 37 GO:0005104 0.0347610445
## 38 GO:0005149 0.0347610445
## 39 GO:0005328 0.0347610445
## 40 GO:0016603 0.0347610445
## 41 GO:0050544 0.0347610445
## 42 GO:1901567 0.0347610445
## 43 GO:0004197 0.0385200354
## 44 GO:0004872 0.0407201611
## 45 GO:0004888 0.0453467861
## 46 GO:0005539 0.0488510844
##                                                           Term
## 1                                         transporter activity
## 2                                          fibronectin binding
## 3                                           chemokine activity
## 4                                         proteoglycan binding
## 5                                     calcium channel activity
## 6                                              heparin binding
## 7  divalent inorganic cation transmembrane transporter activity
## 8                                         ion channel activity
## 9                                             channel activity
## 10               metal ion transmembrane transporter activity
## 11                               voltage-gated channel activity
## 12       substrate-specific transmembrane transporter activity
## 13                                   chloride channel activity
## 14                                             collagen binding
## 15                  cation transmembrane transporter activity
## 16                                        glycoprotein binding
## 17          inorganic anion transmembrane transporter activity
## 18                        voltage-gated cation channel activity
## 19         G-protein coupled chemoattractant receptor activity
## 20                                  guanylate cyclase activity
## 21                                  lipoprotein lipase activity
## 22            phosphoenolpyruvate carboxykinase (GTP) activity
## 23                            proline:sodium symporter activity
## 24                         cation:amino acid symporter activity
## 25                  high voltage-gated calcium channel activity
## 26                                  D-aspartate oxidase activity
## 27                            C-X-C chemokine receptor activity
## 28                                             syntaxin binding
## 29                                           telethonin binding
## 30                              CCR1 chemokine receptor binding
## 31                                   myosin light chain binding
## 32                                       apolipoprotein binding
## 33                                    cytokine receptor binding
## 34                                            lysozyme activity
## 35                            carbonyl reductase (NADPH) activity
```

```
## 36                                  GABA-A receptor activity
## 37                   fibroblast growth factor receptor binding
## 38                             interleukin-1 receptor binding
## 39                  neurotransmitter:sodium symporter activity
## 40                glutaminyl-peptide cyclotransferase activity
## 41                                  arachidonic acid binding
## 42                              fatty acid derivative binding
## 43                      cysteine-type endopeptidase activity
## 44                                          receptor activity
## 45                  transmembrane signaling receptor activity
## 46                                 glycosaminoglycan binding
```

GO enrichment analysis for **CC**

```
ontology(paraGO) <- "CC"
CC <- hyperGTest(paraGO)
summary(CC)[,c(1,2,7)]
```

```
##        GOCCID      Pvalue                                  Term
## 1  GO:0016021 0.005045329         integral component of membrane
## 2  GO:0034707 0.005182821                chloride channel complex
## 3  GO:0005773 0.006792866                                 vacuole
## 4  GO:0005886 0.007282896                         plasma membrane
## 5  GO:0005576 0.008060376                    extracellular region
## 6  GO:0005764 0.011217919                                lysosome
## 7  GO:1902495 0.014224360        transmembrane transporter complex
## 8  GO:0042613 0.019207317              MHC class II protein complex
## 9  GO:0042627 0.019207317                             chylomicron
## 10 GO:0070044 0.019207317 synaptobrevin 2-SNAP-25-syntaxin-1a complex
## 11 GO:0044459 0.022374587                     plasma membrane part
## 12 GO:0005578 0.030738637        proteinaceous extracellular matrix
## 13 GO:0009986 0.031627823                            cell surface
## 14 GO:0005615 0.036921117                     extracellular space
## 15 GO:0005891 0.038051458       voltage-gated calcium channel complex
## 16 GO:0042629 0.038051458                        mast cell granule
## 17 GO:0005777 0.048355996                              peroxisome
```

5. MeSH enrichment analysis

Then, we perform a MeSH ORA for the category **Chemicals and Drugs** by setting 'category="D"'.

```
library(meshr)
library(MeSH.db)
library("org.MeSH.Bta.db")
meshParams <- new("MeSHHyperGParams", geneIds = my.geneID2.org[,2],
                  universeGeneIds = my.univ.geneID2.org[,2],
                  annotation = "org.MeSH.Bta.db", category = "D",
                  database = "gene2pubmed", pvalueCutoff = 0.05, pAdjust = "none")
meshR <- meshHyperGTest(meshParams)
summary(meshR)[!duplicated(summary(meshR)[,7]),c(1,2,7)]
```

```
##       MESHID       Pvalue
```

```
## 5525 D053829 0.0004297762
## 3129 D011971 0.0004484468
## 5625 D055607 0.0005604262
## 4132 D015703 0.0007378416
## 1420 D005136 0.0008895950
## 1725 D007371 0.0009030941
## 3874 D012313 0.0009051932
## 4723 D018121 0.0016549196
## 4711 D018118 0.0032580471
## 4828 D019041 0.0032580471
## 5075 D040281 0.0039522766
## 2477 D009418 0.0048546773
## 1088 D002403 0.0053452724
## 1708 D006684 0.0053452724
## 1807 D007378 0.0053452724
## 2936 D011257 0.0063921646
## 4920 D036341 0.0065851885
## 1612 D006133 0.0070063374
## 4265 D016212 0.0073567061
## 3119 D011759 0.0078929281
## 4120 D013859 0.0078929281
## 4663 D017027 0.0078929281
## 4745 D018123 0.0078929281
## 5181 D042501 0.0078929281
## 4586 D016692 0.0108781888
## 4685 D017468 0.0108781888
## 5509 D053779 0.0108781888
## 4426 D016326 0.0121056751
## 1115 D002786 0.0142790447
## 2431 D009414 0.0142790447
## 3188 D011994 0.0146301601
## 27   D000939 0.0164406947
## 5109 D040301 0.0184670642
## 1947 D008565 0.0201170561
## 1    D000116 0.0237255531
## 10   D000485 0.0237255531
## 21   D000631 0.0237255531
## 23   D000683 0.0237255531
## 25   D000907 0.0237255531
## 83   D000949 0.0237255531
## 142  D000953 0.0237255531
## 145  D001053 0.0237255531
## 157  D001056 0.0237255531
## 197  D001425 0.0237255531
## 202  D001571 0.0237255531
## 610  D002273 0.0237255531
## 1113 D002738 0.0237255531
## 1144 D002914 0.0237255531
## 1399 D004205 0.0237255531
## 1611 D005466 0.0237255531
## 1695 D006632 0.0237255531
## 1699 D006683 0.0237255531
## 1724 D006711 0.0237255531
## 1821 D007653 0.0237255531
```

```
## 2529 D009496 0.0237255531
## 2535 D009961 0.0237255531
## 3112 D011458 0.0237255531
## 4127 D014304 0.0237255531
## 4240 D015846 0.0237255531
## 4243 D015847 0.0237255531
## 4254 D015922 0.0237255531
## 4257 D016028 0.0237255531
## 4259 D016169 0.0237255531
## 4585 D016623 0.0237255531
## 4703 D017476 0.0237255531
## 4706 D017981 0.0237255531
## 4739 D018122 0.0237255531
## 4783 D018679 0.0237255531
## 4784 D018801 0.0237255531
## 4818 D018926 0.0237255531
## 4866 D019323 0.0237255531
## 4873 D019881 0.0237255531
## 4875 D019908 0.0237255531
## 4881 D020098 0.0237255531
## 4911 D024061 0.0237255531
## 4915 D027982 0.0237255531
## 5066 D037521 0.0237255531
## 5178 D040881 0.0237255531
## 5208 D050480 0.0237255531
## 5209 D050705 0.0237255531
## 5263 D050998 0.0237255531
## 5342 D051856 0.0237255531
## 5343 D051906 0.0237255531
## 5357 D051928 0.0237255531
## 5359 D051940 0.0237255531
## 5372 D052218 0.0237255531
## 5422 D053304 0.0237255531
## 5431 D053493 0.0237255531
## 5436 D053707 0.0237255531
## 5593 D054370 0.0237255531
## 5597 D054426 0.0237255531
## 5636 D055654 0.0237255531
## 5642 D055655 0.0237255531
## 5650 D056655 0.0237255531
## 5652 D059866 0.0237255531
## 4841 D019253 0.0267667853
## 5602 D055415 0.0267667853
## 612  D002352 0.0311028987
## 5018 D037181 0.0316253540
## 207  D002135 0.0323346420
## 1263 D003488 0.0323346420
## 2539 D010455 0.0362125161
## 1831 D008070 0.0363565401
## 1401 D004338 0.0368008320
## 4884 D020672 0.0368008320
## 5265 D051197 0.0368008320
## 1146 D003094 0.0394363350
## 5373 D052247 0.0422755912
```

```
## 2    D000276 0.0468906800
## 151  D001054 0.0468906800
## 203  D002097 0.0468906800
## 602  D002217 0.0468906800
## 1822 D008049 0.0468906800
## 1924 D008071 0.0468906800
## 1940 D008079 0.0468906800
## 2910 D010740 0.0468906800
## 2919 D010928 0.0468906800
## 3113 D011464 0.0468906800
## 4128 D015054 0.0468906800
## 4262 D016201 0.0468906800
## 4781 D018336 0.0468906800
## 4787 D018821 0.0468906800
## 4801 D018925 0.0468906800
## 4820 D018996 0.0468906800
## 4868 D019363 0.0468906800
## 4907 D020866 0.0468906800
## 5068 D039481 0.0468906800
## 5189 D043925 0.0468906800
## 5202 D045726 0.0468906800
## 5210 D050799 0.0468906800
## 5260 D050804 0.0468906800
## 5340 D051784 0.0468906800
## 5346 D051920 0.0468906800
## 5367 D052118 0.0468906800
## 5451 D053764 0.0468906800
## 5542 D054340 0.0468906800
## 5555 D054341 0.0468906800
## 5562 D054342 0.0468906800
## 5572 D054345 0.0468906800
## 5579 D054349 0.0468906800
## 5586 D054360 0.0468906800
## 5656 D062367 0.0468906800
## 5659 D064547 0.0468906800
## 165  D001324 0.0480326549
## 5456 D053773 0.0480326549
##                                              MESHTERM
## 5525            Amyloid Precursor Protein Secretases
## 3129                          Receptors, Immunologic
## 5625                Receptors, Natural Killer Cell
## 4132                                     Antigens, CD
## 1420                                     Eye Proteins
## 1725                                 Interferon-gamma
## 3874                                              RNA
## 4723                              Receptors, Cytokine
## 4711                                 Chloride Channels
## 4828                                       L-Selectin
## 5075      Vascular Endothelial Growth Factor Receptor-1
## 2477                                     S100 Proteins
## 1088                                        Cathepsins
## 1708                                    HLA-DR Antigens
## 1807                                     Interleukins
## 2936                                Pregnancy Proteins
```

```
## 4920       Intercellular Signaling Peptides and Proteins
## 1612                                   Growth Substances
## 4265                    Transforming Growth Factor beta
## 3119                                         Pyrrolidines
## 4120                                       Thiocarbamates
## 4663                      Protein Tyrosine Phosphatases
## 4745                               Receptors, Interleukin
## 5181                                  Angiogenic Proteins
## 4586             Receptors, Antigen, T-Cell, gamma-delta
## 4685                 Receptors, Fibroblast Growth Factor
## 5509                    Latent TGF-beta Binding Proteins
## 4426                      Extracellular Matrix Proteins
## 1115              Cholesterol Side-Chain Cleavage Enzyme
## 2431                                Nerve Growth Factors
## 3188                                Recombinant Proteins
## 27                                              Epitopes
## 5109       Vascular Endothelial Growth Factor Receptor-2
## 1947                                   Membrane Proteins
## 1                                  Acetylgalactosamine
## 10                                             Allergens
## 21                                        Aminopyridines
## 23                              Serum Amyloid P-Component
## 25                                  Antibodies, Bacterial
## 83                 Histocompatibility Antigens Class II
## 142                                  Antigens, Protozoan
## 145                                      Apolipoproteins
## 157                                    Apolipoproteins C
## 197                      Bacterial Outer Membrane Proteins
## 202                                         Benzoflavones
## 610                                          Carcinogens
## 1113                                          Chloroquine
## 1144                                         Chylomicrons
## 1399                                       Cromolyn Sodium
## 1611                                        Fluorocarbons
## 1695                                            Histamine
## 1699                                       HLA-DQ Antigens
## 1724                                          Homocystine
## 1821                                     Ketocholesterols
## 2529                                          Neurotensin
## 2535                                          Orosomucoid
## 3112                                     Prostaglandins E
## 4127                                             Triolein
## 4240                                            Monokines
## 4243                                        Interleukin-4
## 4254                                        Complement C1q
## 4257                       Receptors, Leukocyte-Adhesion
## 4259           Lymphocyte Function-Associated Antigen-1
## 4585                                            Ion Pumps
## 4703                            Receptors, Neuropeptide Y
## 4706                          Receptors, Neurotransmitter
## 4739                                        Antigens, CD80
## 4783                                  Cholinergic Agonists
## 4784                                         Antigens, CD2
## 4818                                  Anti-Allergic Agents
```

```
## 4866                           omega-N-Methylarginine
## 4873                           Aminoacyltransferases
## 4875                     Proto-Oncogene Proteins c-raf
## 4881                       Natriuretic Peptide, C-Type
## 4911                                Collagen Type III
## 4915                     Sodium-Bicarbonate Symporters
## 5066                                Virulence Factors
## 5178                                  Antigens, CD11a
## 5208 Plasma Membrane Neurotransmitter Transport Proteins
## 5209                Receptors, Guanylate Cyclase-Coupled
## 5263          bcl-2 Homologous Antagonist-Killer Protein
## 5342        Fanconi Anemia Complementation Group Proteins
## 5343                                     Smad7 Protein
## 5357                                    Antigens, CD47
## 5359                                    Antigens, CD86
## 5372       Fanconi Anemia Complementation Group C Protein
## 5422                               Apolipoprotein C-II
## 5431                   Cholestanetriol 26-Monooxygenase
## 5436                         Receptors, Interleukin-12
## 5593                                    Chemokine CXCL9
## 5597                                    Chemokine CXCL2
## 5636            NK Cell Lectin-Like Receptor Subfamily C
## 5642            NK Cell Lectin-Like Receptor Subfamily K
## 5650                                        Cathepsin H
## 5652                                 HLA-DQ beta-Chains
## 4841                      Proto-Oncogene Proteins c-bcl-2
## 5602                        Bone Morphogenetic Protein 4
## 612                                    Carrier Proteins
## 5018                                    Lectins, C-Type
## 207                            Calcium-Binding Proteins
## 1263                                   Cyanogen Bromide
## 2539                                           Peptides
## 1831                                Lipopolysaccharides
## 1401                                  Drug Combinations
## 4884                                  I-kappa B Proteins
## 5265                                 Toll-Like Receptor 4
## 1146                                           Collagen
## 5373                      Nitric Oxide Synthase Type II
## 2                               Adjuvants, Immunologic
## 151                                  Apolipoproteins A
## 203                                   C-Reactive Protein
## 602                                            Carbachol
## 1822                                              Lipase
## 1924                                   Lipoprotein Lipase
## 1940                                   Lipoproteins, VLDL
## 2910                                      Phospholipases
## 2919                                   Placental Lactogen
## 3113                                        Epoprostenol
## 4128                                             Zymosan
## 4262                       Receptors, Lymphocyte Homing
## 4781                        Receptors, Aryl Hydrocarbon
## 4787                                      Antigens, CD18
## 4801                                          Chemokines
## 4820                                     Myelin P2 Protein
```

```
## 4868                          Cytochrome P-450 CYP1A1
## 4907                             omega-Conotoxin GVIA
## 5068                                  Antigens, CD11b
## 5189                      Angiogenesis Inducing Agents
## 5202                                Metalloproteases
## 5210                       STAT5 Transcription Factor
## 5260                              D-Aspartate Oxidase
## 5340      Aryl Hydrocarbon Receptor Nuclear Translocator
## 5346            NK Cell Lectin-Like Receptor Subfamily D
## 5367             Lysosomal-Associated Membrane Protein 1
## 5451                                    Presenilin-1
## 5542                                  Receptors, KIR
## 5555                              Receptors, KIR3DL1
## 5562                              Receptors, KIR2DL1
## 5572                              Receptors, KIR2DL4
## 5579                              Receptors, KIR3DS1
## 5586                                 Chemokine CXCL1
## 5656                            ortho-Aminobenzoates
## 5659                      Myxovirus Resistance Proteins
## 165                                     Autoantigens
## 5456                     Transforming Growth Factor beta1
```

Switching to a different category is easily done by the 'category<-' function. Here, we use **Diseases** (category = "C").

```
category(meshParams) <- "C"
meshR <- meshHyperGTest(meshParams)
summary(meshR)[!duplicated(summary(meshR)[,7]),c(1,2,7)]
```

```
##       MESHID       Pvalue                              MESHTERM
## 864  D008414 3.250507e-05                      Mastitis, Bovine
## 820  D005334 1.654920e-03                                 Fever
## 75   D002418 5.757735e-03                      Cattle Diseases
## 1192 D010283 1.087819e-02                    Paratuberculosis
## 1318 D014380 1.087819e-02                   Tuberculosis, Bovine
## 1    D000034 2.372555e-02                  Abortion, Veterinary
## 69   D002007 2.372555e-02                    Brucellosis, Bovine
## 817  D003092 2.372555e-02                               Colitis
## 818  D003320 2.372555e-02                         Corneal Ulcer
## 819  D005199 2.372555e-02                        Fanconi Anemia
## 823  D007662 2.372555e-02                              Ketosis
## 824  D007794 2.372555e-02                      Lameness, Animal
## 855  D008268 2.372555e-02                 Macular Degeneration
## 1287 D010922 2.372555e-02                    Placenta Diseases
## 1288 D011248 2.372555e-02               Pregnancy Complications
## 1316 D012766 2.372555e-02             Pasteurellosis, Pneumonic
## 1341 D015228 2.372555e-02                  Hypertriglyceridemia
## 1343 D015658 2.372555e-02                        HIV Infections
## 1344 D018370 2.372555e-02 Leukocyte-Adhesion Deficiency Syndrome
## 1651 D020964 2.372555e-02                           Embryo Loss
## 857  D008382 4.689068e-02                       Marfan Syndrome
## 1348 D020022 4.803265e-02      Genetic Predisposition to Disease
```

MeSH ORA for **Anatomy** (category = "A").

```r
category(meshParams) <- "A"
meshR <- meshHyperGTest(meshParams)
summary(meshR)[!duplicated(summary(meshR)[,7]),c(1,2,7)]
```

```
##        MESHID       Pvalue                        MESHTERM
## 4202 D009504 4.891771e-06                      Neutrophils
## 4831 D012867 4.484468e-04                             Skin
## 1610 D008892 4.601818e-04                             Milk
## 62   D000653 5.604262e-04                   Amniotic Fluid
## 5047 D014822 5.604262e-04                    Vitreous Body
## 73   D002460 1.156952e-03                        Cell Line
## 4828 D012708 1.654920e-03                   Sertoli Cells
## 4918 D014132 4.854677e-03                          Trachea
## 1469 D007908 5.871500e-03               Lens, Crystalline
## 1297 D007668 7.562931e-03                           Kidney
## 5123 D019169 1.087819e-02                    Jurkat Cells
## 869  D002462 1.862338e-02                    Cell Membrane
## 5200 D042783 1.894443e-02               Endothelial Cells
## 1552 D008264 2.062578e-02                     Macrophages
## 4611 D012160 2.110818e-02                           Retina
## 4560 D010902 2.224343e-02                  Pituitary Gland
## 5052 D016176 2.224343e-02            T-Lymphocyte Subsets
## 5136 D020419 2.224343e-02 Photoreceptor Cells, Vertebrate
## 58   D000482 2.372555e-02                       Allantois
## 1204 D002883 2.372555e-02     Chromosomes, Human, Pair 14
## 1247 D003319 2.372555e-02                  Corneal Stroma
## 1262 D005243 2.372555e-02                           Feces
## 1551 D008125 2.372555e-02                  Locus Coeruleus
## 4917 D013666 2.372555e-02                           Tears
## 5616 D050824 2.372555e-02                   Carpal Joints
## 4285 D009865 2.408291e-02                         Oocytes
## 844  D002461 3.680083e-02          Cell Line, Transformed
## 1214 D003126 4.227559e-02                       Colostrum
## 4157 D009000 4.227559e-02                       Monocytes
## 5158 D021962 4.227559e-02           Membrane Microdomains
## 68   D001933 4.689068e-02                      Brain Stem
## 1198 D002823 4.689068e-02                         Chorion
## 1207 D002894 4.689068e-02      Chromosomes, Human, Pair 4
## 1248 D004817 4.689068e-02                       Epidermis
## 1292 D005920 4.689068e-02             Glomerular Mesangium
## 1447 D007694 4.689068e-02           Killer Cells, Natural
## 1546 D007985 4.689068e-02                    Leydig Cells
## 1607 D008648 4.689068e-02                        Mesoderm
## 4199 D009457 4.689068e-02                       Neuroglia
## 4557 D010586 4.689068e-02                     Phagocytes
## 4821 D012463 4.689068e-02                          Saliva
## 1    D000302 4.803265e-02                  Adrenal Cortex
## 4953 D014327 4.943893e-02                    Trophoblasts
```

MeSH ORA for **Phenomena and Processes** (category = “G”).

```
category(meshParams) <- "G"
meshR <- meshHyperGTest(meshParams)
summary(meshR)[!duplicated(summary(meshR)[,7]),c(1,2,7)]
```

```
##          MESHID      Pvalue                                    MESHTERM
## 1843  D000595 0.001667036                          Amino Acid Sequence
## 17567 D018375 0.003258047                        Neutrophil Activation
## 5     D000483 0.004055151                                      Alleles
## 13517 D010802 0.005081324                                    Phylogeny
## 11769 D007774 0.005360661                                    Lactation
## 16289 D017386 0.006583581                Sequence Homology, Amino Acid
## 15745 D014162 0.007807192                                 Transfection
## 6853  D002633 0.007892928                                   Chemotaxis
## 7273  D005838 0.008071564                                     Genotype
## 6894  D005810 0.010280847                             Multigene Family
## 16205 D016922 0.010878189                                   Cell Aging
## 11554 D006031 0.018469771                                Glycosylation
## 11686 D006720 0.022243427                                   Homozygote
## 14818 D011270 0.022917140                            Pregnancy, Animal
## 6884  D002883 0.023725553                 Chromosomes, Human, Pair 14
## 14813 D011232 0.023725553                     Chemical Precipitation
## 16194 D015321 0.023725553                          Gene Rearrangement
## 18132 D047108 0.024817577                        Embryonic Development
## 17573 D018919 0.025341189           Neovascularization, Physiologic
## 6253  D002452 0.026766785                                   Cell Count
## 16213 D017384 0.039436335                            Sequence Deletion
## 17969 D020816 0.041048172                            Amino Acid Motifs
## 13478 D008213 0.042275591                        Lymphocyte Activation
## 14047 D011110 0.043060921                        Polymorphism, Genetic
## 15380 D014158 0.044402394                       Transcription, Genetic
## 14947 D012150 0.045979017 Polymorphism, Restriction Fragment Length
## 1     D000220 0.046890680                      Adaptation, Biological
## 6242  D001770 0.046890680                  Blood Bactericidal Activity
## 6887  D002894 0.046890680                   Chromosomes, Human, Pair 4
## 17549 D017951 0.046890680                        Antigen Presentation
## 17967 D020131 0.046890680                            Genes, Duplicate
## 17664 D020022 0.048032655            Genetic Predisposition to Disease
## 15177 D013329 0.049061161              Structure-Activity Relationship
```

6. Session Information

```
sessionInfo()
```

```
## R version 3.1.2 (2014-10-31)
## Platform: x86_64-apple-darwin13.4.0 (64-bit)
##
## locale:
## [1] en_US.UTF-8/en_US.UTF-8/en_US.UTF-8/C/en_US.UTF-8/en_US.UTF-8
##
## attached base packages:
##  [1] grid      parallel  stats4    stats     graphics  grDevices utils
```

```
## [8] datasets  methods   base
##
## other attached packages:
##  [1] org.MeSH.Bta.db_1.2.0     meshr_1.2.2
##  [3] org.MeSH.Syn.db_1.2.0     org.MeSH.Bsu.168.db_1.2.0
##  [5] org.MeSH.Atu.K84.db_1.2.0 org.MeSH.Aca.db_1.2.0
##  [7] org.MeSH.Hsa.db_1.2.0     MeSH.PCR.db_1.2.0
##  [9] MeSH.AOR.db_1.2.0         MeSH.db_1.2.0
## [11] MeSHDbi_1.2.0             org.Hs.eg.db_3.0.0
## [13] cummeRbund_2.8.2          Gviz_1.10.2
## [15] rtracklayer_1.26.2        GenomicRanges_1.18.3
## [17] fastcluster_1.1.13        reshape2_1.4
## [19] ggplot2_1.0.0             fdrtool_1.2.13
## [21] GOstats_2.32.0            graph_1.44.0
## [23] Category_2.32.0           GO.db_3.0.0
## [25] Matrix_1.1-4              org.Bt.eg.db_3.0.0
## [27] RSQLite_1.0.0             DBI_0.3.1
## [29] AnnotationDbi_1.28.1      GenomeInfoDb_1.2.3
## [31] IRanges_2.0.0             S4Vectors_0.4.0
## [33] Biobase_2.26.0            BiocGenerics_0.12.1
##
## loaded via a namespace (and not attached):
##  [1] acepack_1.3-3.3          annotate_1.44.0
##  [3] AnnotationForge_1.8.1    base64enc_0.1-2
##  [5] BatchJobs_1.5            BBmisc_1.8
##  [7] BiocParallel_1.0.0       biomaRt_2.22.0
##  [9] Biostrings_2.34.0        biovizBase_1.14.0
## [11] bitops_1.0-6             brew_1.0-6
## [13] BSgenome_1.34.0          checkmate_1.5.0
## [15] cluster_1.15.3           codetools_0.2-9
## [17] colorspace_1.2-4         dichromat_2.0-0
## [19] digest_0.6.4             evaluate_0.5.5
## [21] fail_1.2                 foreach_1.4.2
## [23] foreign_0.8-61           formatR_1.0
## [25] Formula_1.1-2            genefilter_1.48.1
## [27] GenomicAlignments_1.2.1  GenomicFeatures_1.18.2
## [29] GSEABase_1.28.0          gtable_0.1.2
## [31] Hmisc_3.14-5             htmltools_0.2.6
## [33] iterators_1.0.7          knitr_1.8
## [35] lattice_0.20-29          latticeExtra_0.6-26
## [37] MASS_7.3-35              matrixStats_0.10.3
## [39] munsell_0.4.2            nnet_7.3-8
## [41] plyr_1.8.1               proto_0.3-10
## [43] R.methodsS3_1.6.1        RBGL_1.42.0
## [45] RColorBrewer_1.0-5       Rcpp_0.11.3
## [47] RCurl_1.95-4.3           rmarkdown_0.3.10
## [49] rpart_4.1-8              Rsamtools_1.18.2
## [51] scales_0.2.4             sendmailR_1.2-1
## [53] splines_3.1.2            stringr_0.6.2
## [55] survival_2.37-7          tools_3.1.2
## [57] VariantAnnotation_1.12.4 XML_3.98-1.1
## [59] xtable_1.7-4             XVector_0.6.0
## [61] yaml_2.1.13              zlibbioc_1.12.0
```
