# Supplementary material for: An application of MeSH enrichment analysis in livestock
Source: Anim Genet. 2015 Jun 2;46(4):381–7. doi: 10.1111/age.12307 (PMC5032990; doi:10.1111/age.12307)
Supplement: Supplementary file 2 — Appendix S2 MeSH over‐representation analysis (Swine) [file AGE-46-381-s002.pdf]

APPENDIX S2

MeSH over-representation analysis (Swine)

This is a reproducible report written by an R Markdown.

1. Create a vector of background (universe) genes

We first create a vector of all genes.

```
library(biomaRt)
## access to biomaRt
mart <- useMart(biomart = "ensembl", dataset = "sscrofa_gene_ensembl")
univ.geneID <-getBM(attributes=c("ensembl_gene_id", "entrezgene", "hgnc_symbol"), mart = mart) # 27243
## remove genes with no corresponding Entrez Gene ID
univ.geneID2 <- univ.geneID[!is.na(univ.geneID[,2]),] # 18251
## remove duplicated Entrez Gene ID
univ.geneID3 <- univ.geneID2[ !duplicated(univ.geneID2[,2]),] # 17130
```

2. Create a vector of significant genes

Secondly, we create a vector of significant genes by reading the file *sscGenes.txt*.

```
## read data
genes.ssc <- read.table("sscGenes2.txt", colClasses = c("character", rep("NULL", 5)), fill=TRUE,
                        header=FALSE, stringsAsFactors=FALSE)
colnames(genes.ssc) <- "ensembl_gene_id"
## merge two files
my.geneID <- merge(genes.ssc, univ.geneID3, by ="ensembl_gene_id") # 155
## remove duplicated Entrez Gene ID
my.geneID2 <- my.geneID[ !duplicated(my.geneID$entrezgene),] # 152
```

3. GO enrichment analysis

We perform a GO analysis via the *GOstats* package.

```
library("org.Ss.eg.db")
library("GOstats")
paraGO <- new("GOHyperGParams", geneIds=my.geneID2[,2], universeGeneIds=univ.geneID3[,2],
              annotation="org.Ss.eg.db", ontology="BP", pvalueCutoff=0.05,
              conditional=TRUE, testDirection="over")
```

GO enrichment analysis for **BP**

```
BP <- hyperGTest(paraGO)
summary(BP)[,c(1,2,7)]
```

```
##         GOBPID       Pvalue
## 1   GO:0002125 2.414211e-06
## 2   GO:0070493 2.073360e-05
```

```
## 3   GO:0042631 7.252975e-05
## 4   GO:2000484 7.252975e-05
## 5   GO:0001992 2.163746e-04
## 6   GO:0007621 2.163746e-04
## 7   GO:0031394 2.163746e-04
## 8   GO:0051970 2.163746e-04
## 9   GO:2000778 2.163746e-04
## 10  GO:0032849 4.303350e-04
## 11  GO:0042711 4.303350e-04
## 12  GO:2001279 4.303350e-04
## 13  GO:0006112 4.865599e-04
## 14  GO:0007204 5.818235e-04
## 15  GO:0045819 7.132263e-04
## 16  GO:0060180 7.132263e-04
## 17  GO:0003015 7.462577e-04
## 18  GO:0060986 8.312094e-04
## 19  GO:0006506 9.499953e-04
## 20  GO:0051459 1.040437e-03
## 21  GO:0014049 1.063879e-03
## 22  GO:0035810 1.063879e-03
## 23  GO:0042713 1.063879e-03
## 24  GO:0009251 1.218596e-03
## 25  GO:0044247 1.218596e-03
## 26  GO:0003084 1.481137e-03
## 27  GO:0035025 1.481137e-03
## 28  GO:0048523 1.522633e-03
## 29  GO:0005976 1.597594e-03
## 30  GO:0006664 1.920400e-03
## 31  GO:0035815 1.963859e-03
## 32  GO:0007625 2.510921e-03
## 33  GO:0009415 2.510921e-03
## 34  GO:0043084 2.510921e-03
## 35  GO:2001244 2.510921e-03
## 36  GO:0008272 3.121207e-03
## 37  GO:0015939 3.121207e-03
## 38  GO:0045723 3.121207e-03
## 39  GO:0045907 3.121207e-03
## 40  GO:0046457 3.121207e-03
## 41  GO:0006874 3.137600e-03
## 42  GO:0043270 3.319385e-03
## 43  GO:0042158 3.534758e-03
## 44  GO:0051209 3.534758e-03
## 45  GO:0051282 3.534758e-03
## 46  GO:0034329 3.622341e-03
## 47  GO:0072507 4.522534e-03
## 48  GO:0010460 4.527078e-03
## 49  GO:0008645 4.633205e-03
## 50  GO:0007015 4.767898e-03
## 51  GO:0016053 4.770176e-03
## 52  GO:0008643 6.006432e-03
## 53  GO:0006693 6.172849e-03
## 54  GO:0032892 6.172849e-03
## 55  GO:0035338 6.172849e-03
## 56  GO:0044062 6.172849e-03
```

```
## 57  GO:0051955 6.172849e-03
## 58  GO:0044724 6.518355e-03
## 59  GO:0044705 7.083061e-03
## 60  GO:0006073 7.502866e-03
## 61  GO:0042462 8.050112e-03
## 62  GO:0051412 8.050112e-03
## 63  GO:0031323 8.258403e-03
## 64  GO:0000023 8.564280e-03
## 65  GO:0002534 8.564280e-03
## 66  GO:0002741 8.564280e-03
## 67  GO:0005985 8.564280e-03
## 68  GO:0006408 8.564280e-03
## 69  GO:0009733 8.564280e-03
## 70  GO:0009845 8.564280e-03
## 71  GO:0010804 8.564280e-03
## 72  GO:0035865 8.564280e-03
## 73  GO:0035926 8.564280e-03
## 74  GO:0042450 8.564280e-03
## 75  GO:0043181 8.564280e-03
## 76  GO:0043311 8.564280e-03
## 77  GO:0046833 8.564280e-03
## 78  GO:0050652 8.564280e-03
## 79  GO:0051460 8.564280e-03
## 80  GO:0060588 8.564280e-03
## 81  GO:0070948 8.564280e-03
## 82  GO:0070950 8.564280e-03
## 83  GO:0070961 8.564280e-03
## 84  GO:0070963 8.564280e-03
## 85  GO:0071314 8.564280e-03
## 86  GO:0071638 8.564280e-03
## 87  GO:0072643 8.564280e-03
## 88  GO:0090198 8.564280e-03
## 89  GO:0097029 8.564280e-03
## 90  GO:1900011 8.564280e-03
## 91  GO:2000405 8.564280e-03
## 92  GO:0031644 9.072985e-03
## 93  GO:0051954 9.072985e-03
## 94  GO:0010907 1.015067e-02
## 95  GO:0035337 1.015067e-02
## 96  GO:0046326 1.015067e-02
## 97  GO:0044706 1.092574e-02
## 98  GO:0060401 1.099555e-02
## 99  GO:0035176 1.128218e-02
## 100 GO:0045851 1.128218e-02
## 101 GO:0055078 1.128218e-02
## 102 GO:0010827 1.155903e-02
## 103 GO:0006091 1.182295e-02
## 104 GO:0061564 1.182295e-02
## 105 GO:0044260 1.216307e-02
## 106 GO:0051057 1.246654e-02
## 107 GO:0051238 1.246654e-02
## 108 GO:0002791 1.287311e-02
## 109 GO:0072330 1.287311e-02
## 110 GO:0030154 1.325425e-02
```

```
## 111 GO:0034332 1.334813e-02
## 112 GO:0051453 1.370278e-02
## 113 GO:0055082 1.452474e-02
## 114 GO:0006084 1.498993e-02
## 115 GO:0007618 1.498993e-02
## 116 GO:0014068 1.498993e-02
## 117 GO:1901879 1.498993e-02
## 118 GO:0046474 1.666274e-02
## 119 GO:0048812 1.682329e-02
## 120 GO:0001771 1.705603e-02
## 121 GO:0002248 1.705603e-02
## 122 GO:0006478 1.705603e-02
## 123 GO:0007402 1.705603e-02
## 124 GO:0009438 1.705603e-02
## 125 GO:0010015 1.705603e-02
## 126 GO:0010054 1.705603e-02
## 127 GO:0022622 1.705603e-02
## 128 GO:0034137 1.705603e-02
## 129 GO:0034140 1.705603e-02
## 130 GO:0034141 1.705603e-02
## 131 GO:0034351 1.705603e-02
## 132 GO:0035089 1.705603e-02
## 133 GO:0042256 1.705603e-02
## 134 GO:0043148 1.705603e-02
## 135 GO:0048149 1.705603e-02
## 136 GO:0048765 1.705603e-02
## 137 GO:0048767 1.705603e-02
## 138 GO:0050653 1.705603e-02
## 139 GO:0060252 1.705603e-02
## 140 GO:0060385 1.705603e-02
## 141 GO:0061339 1.705603e-02
## 142 GO:0072608 1.705603e-02
## 143 GO:0097211 1.705603e-02
## 144 GO:2000098 1.705603e-02
## 145 GO:0048667 1.736729e-02
## 146 GO:0070873 1.771326e-02
## 147 GO:0046467 1.810621e-02
## 148 GO:0048699 1.812233e-02
## 149 GO:0050880 1.885329e-02
## 150 GO:0043471 1.914757e-02
## 151 GO:0051592 1.961729e-02
## 152 GO:0032642 2.062912e-02
## 153 GO:0045913 2.062912e-02
## 154 GO:1901570 2.062912e-02
## 155 GO:0030072 2.166283e-02
## 156 GO:0003044 2.215699e-02
## 157 GO:0030004 2.215699e-02
## 158 GO:0060048 2.215699e-02
## 159 GO:0006766 2.284293e-02
## 160 GO:0009311 2.373031e-02
## 161 GO:0005975 2.399894e-02
## 162 GO:0010256 2.401274e-02
## 163 GO:0044262 2.524357e-02
## 164 GO:0046883 2.524357e-02
```

```
## 165 GO:0070830 2.534820e-02
## 166 GO:0071616 2.534820e-02
## 167 GO:0002086 2.547586e-02
## 168 GO:0002888 2.547586e-02
## 169 GO:0006434 2.547586e-02
## 170 GO:0006975 2.547586e-02
## 171 GO:0030836 2.547586e-02
## 172 GO:0032930 2.547586e-02
## 173 GO:0045197 2.547586e-02
## 174 GO:0048865 2.547586e-02
## 175 GO:0050774 2.547586e-02
## 176 GO:0060022 2.547586e-02
## 177 GO:0060100 2.547586e-02
## 178 GO:0070286 2.547586e-02
## 179 GO:0090594 2.547586e-02
## 180 GO:1902566 2.547586e-02
## 181 GO:0044272 2.634016e-02
## 182 GO:0046488 2.634016e-02
## 183 GO:0045055 2.700981e-02
## 184 GO:0019318 2.781812e-02
## 185 GO:0032677 2.871429e-02
## 186 GO:0046530 2.871429e-02
## 187 GO:0015833 3.124993e-02
## 188 GO:0045017 3.235683e-02
## 189 GO:0048666 3.301916e-02
## 190 GO:0032535 3.311166e-02
## 191 GO:0051705 3.316839e-02
## 192 GO:0051129 3.342271e-02
## 193 GO:0010642 3.382437e-02
## 194 GO:0014012 3.382437e-02
## 195 GO:0021554 3.382437e-02
## 196 GO:0030432 3.382437e-02
## 197 GO:0034145 3.382437e-02
## 198 GO:0048539 3.382437e-02
## 199 GO:0051895 3.382437e-02
## 200 GO:0060287 3.382437e-02
## 201 GO:2000144 3.382437e-02
## 202 GO:2000310 3.382437e-02
## 203 GO:2000353 3.382437e-02
## 204 GO:0002367 3.407657e-02
## 205 GO:0021675 3.407657e-02
## 206 GO:0050707 3.407657e-02
## 207 GO:0048589 3.492128e-02
## 208 GO:0032880 3.521664e-02
## 209 GO:0044255 3.566352e-02
## 210 GO:0044057 3.724675e-02
## 211 GO:0019432 3.785067e-02
## 212 GO:0030029 3.900379e-02
## 213 GO:0003014 3.979510e-02
## 214 GO:0007202 3.979510e-02
## 215 GO:0010923 3.979510e-02
## 216 GO:0030193 3.979510e-02
## 217 GO:0046460 3.979510e-02
## 218 GO:1900274 3.979510e-02
```

```
## 219 GO:2001233 3.979510e-02
## 220 GO:0032990 4.049854e-02
## 221 GO:0080090 4.117167e-02
## 222 GO:0045823 4.121869e-02
## 223 GO:0031344 4.129751e-02
## 224 GO:0071356 4.177676e-02
## 225 GO:0072659 4.177676e-02
## 226 GO:0002278 4.210215e-02
## 227 GO:0002447 4.210215e-02
## 228 GO:0006527 4.210215e-02
## 229 GO:0006684 4.210215e-02
## 230 GO:0031579 4.210215e-02
## 231 GO:0050655 4.210215e-02
## 232 GO:0051709 4.210215e-02
## 233 GO:0051873 4.210215e-02
## 234 GO:0061099 4.210215e-02
## 235 GO:0061245 4.210215e-02
## 236 GO:0090224 4.210215e-02
## 237 GO:0008217 4.302025e-02
## 238 GO:0031960 4.302025e-02
## 239 GO:0040011 4.367228e-02
## 240 GO:0051146 4.420330e-02
## 241 GO:0055114 4.439665e-02
## 242 GO:2000021 4.535415e-02
## 243 GO:0002683 4.540255e-02
## 244 GO:0009914 4.648659e-02
## 245 GO:0008152 4.660552e-02
## 246 GO:0032956 4.661793e-02
## 247 GO:0043436 4.723082e-02
## 248 GO:0030003 4.742850e-02
##                                                                           Term
## 1                                                   maternal aggressive behavior
## 2                                              thrombin receptor signaling pathway
## 3                                           cellular response to water deprivation
## 4                                    positive regulation of interleukin-8 secretion
## 5                   regulation of systemic arterial blood pressure by vasopressin
## 6                                      negative regulation of female receptivity
## 7                       positive regulation of prostaglandin biosynthetic process
## 8                          negative regulation of transmission of nerve impulse
## 9                                    positive regulation of interleukin-6 secretion
## 10                                 positive regulation of cellular pH reduction
## 11                                                             maternal behavior
## 12                      regulation of unsaturated fatty acid biosynthetic process
## 13                                             energy reserve metabolic process
## 14                     positive regulation of cytosolic calcium ion concentration
## 15                             positive regulation of glycogen catabolic process
## 16                                                        female mating behavior
## 17                                                                 heart process
## 18                                                    endocrine hormone secretion
## 19                                                GPI anchor biosynthetic process
## 20                                          regulation of corticotropin secretion
## 21                                     positive regulation of glutamate secretion
## 22                                          positive regulation of urine volume
## 23                                                              sperm ejaculation
```

```
## 24                                                                glucan catabolic process
## 25                                               cellular polysaccharide catabolic process
## 26                                   positive regulation of systemic arterial blood pressure
## 27                                    positive regulation of Rho protein signal transduction
## 28                                                 negative regulation of cellular process
## 29                                                        polysaccharide metabolic process
## 30                                                            glycolipid metabolic process
## 31                                            positive regulation of renal sodium excretion
## 32                                                                        grooming behavior
## 33                                                                        response to water
## 34                                                                           penile erection
## 35                              positive regulation of intrinsic apoptotic signaling pathway
## 36                                                                        sulfate transport
## 37                                                          pantothenate metabolic process
## 38                                   positive regulation of fatty acid biosynthetic process
## 39                                                  positive regulation of vasoconstriction
## 40                                                        prostanoid biosynthetic process
## 41                                                         cellular calcium ion homeostasis
## 42                                                      positive regulation of ion transport
## 43                                                         lipoprotein biosynthetic process
## 44                                          release of sequestered calcium ion into cytosol
## 45                                                 regulation of sequestering of calcium ion
## 46                                                                    cell junction assembly
## 47                                                     divalent inorganic cation homeostasis
## 48                                                        positive regulation of heart rate
## 49                                                                          hexose transport
## 50                                                               actin filament organization
## 51                                                         organic acid biosynthetic process
## 52                                                                    carbohydrate transport
## 53                                                          prostaglandin metabolic process
## 54                                            positive regulation of organic acid transport
## 55                                             long-chain fatty-acyl-CoA biosynthetic process
## 56                                                                  regulation of excretion
## 57                                                     regulation of amino acid transport
## 58                                             single-organism carbohydrate catabolic process
## 59                                                      multi-organism reproductive behavior
## 60                                                         cellular glucan metabolic process
## 61                                                     eye photoreceptor cell development
## 62                                                               response to corticosterone
## 63                                                  regulation of cellular metabolic process
## 64                                                                maltose metabolic process
## 65                                  cytokine production involved in inflammatory response
## 66                     positive regulation of cytokine secretion involved in immune response
## 67                                                                sucrose metabolic process
## 68                                                                snRNA export from nucleus
## 69                                                                        response to auxin
## 70                                                                          seed germination
## 71                negative regulation of tumor necrosis factor-mediated signaling pathway
## 72                                                      cellular response to potassium ion
## 73                                                  chemokine (C-C motif) ligand 2 secretion
## 74                                                arginine biosynthetic process via ornithine
## 75                                                                     vacuolar sequestering
## 76                                            positive regulation of eosinophil degranulation
## 77                                             positive regulation of RNA export from nucleus
```

```
## 78     dermatan sulfate proteoglycan biosynthetic process, polysaccharide chain biosynthetic process
## 79                                        negative regulation of corticotropin secretion
## 80                                    negative regulation of lipoprotein lipid oxidation
## 81                                        regulation of neutrophil mediated cytotoxicity
## 82                                  regulation of neutrophil mediated killing of bacterium
## 83                       positive regulation of neutrophil mediated killing of symbiont cell
## 84          positive regulation of neutrophil mediated killing of gram-negative bacterium
## 85                                                              cellular response to cocaine
## 86                     negative regulation of monocyte chemotactic protein-1 production
## 87                                                               interferon-gamma secretion
## 88                                            negative regulation of chemokine secretion
## 89                                                   mature dendritic cell differentiation
## 90          negative regulation of corticotropin-releasing hormone receptor activity
## 91                                             negative regulation of T cell migration
## 92                                              regulation of neurological system process
## 93                                                positive regulation of amine transport
## 94                                     positive regulation of glucose metabolic process
## 95                                                       fatty-acyl-CoA metabolic process
## 96                                                 positive regulation of glucose import
## 97                                                  multi-multicellular organism process
## 98                                                      cytosolic calcium ion transport
## 99                                                                        social behavior
## 100                                                                         pH reduction
## 101                                                                sodium ion homeostasis
## 102                                                        regulation of glucose transport
## 103                                     generation of precursor metabolites and energy
## 104                                                                      axon development
## 105                                                cellular macromolecule metabolic process
## 106                       positive regulation of small GTPase mediated signal transduction
## 107                                                              sequestering of metal ion
## 108                                                          regulation of peptide secretion
## 109                                               monocarboxylic acid biosynthetic process
## 110                                                                  cell differentiation
## 111                                                          adherens junction organization
## 112                                                         regulation of intracellular pH
## 113                                                          cellular chemical homeostasis
## 114                                                            acetyl-CoA metabolic process
## 115                                                                                 mating
## 116                     positive regulation of phosphatidylinositol 3-kinase signaling
## 117                                                 regulation of protein depolymerization
## 118                                               glycerophospholipid biosynthetic process
## 119                                                         neuron projection morphogenesis
## 120                                                         immunological synapse formation
## 121         connective tissue replacement involved in inflammatory response wound healing
## 122                                                               peptidyl-tyrosine sulfation
## 123                                                  ganglion mother cell fate determination
## 124                                                          methylglyoxal metabolic process
## 125                                                                     root morphogenesis
## 126                                                              trichoblast differentiation
## 127                                                                 root system development
## 128                       positive regulation of toll-like receptor 2 signaling pathway
## 129                       negative regulation of toll-like receptor 3 signaling pathway
## 130                       positive regulation of toll-like receptor 3 signaling pathway
## 131                                      negative regulation of glial cell apoptotic process
```

```
## 132                                          establishment of apical/basal cell polarity
## 133                                                              mature ribosome assembly
## 134                                                          mitotic spindle stabilization
## 135                                                          behavioral response to ethanol
## 136                                                          root hair cell differentiation
## 137                                                                    root hair elongation
## 138 chondroitin sulfate proteoglycan biosynthetic process, polysaccharide chain biosynthetic process
## 139                                          positive regulation of glial cell proliferation
## 140                                                  axonogenesis involved in innervation
## 141                                  establishment or maintenance of monopolar cell polarity
## 142                                                                interleukin-10 secretion
## 143                                      cellular response to gonadotropin-releasing hormone
## 144                                negative regulation of smooth muscle cell-matrix adhesion
## 145                                     cell morphogenesis involved in neuron differentiation
## 146                                                 regulation of glycogen metabolic process
## 147                                                     membrane lipid biosynthetic process
## 148                                                                   generation of neurons
## 149                                                          regulation of blood vessel size
## 150                                     regulation of cellular carbohydrate catabolic process
## 151                                                                 response to calcium ion
## 152                                                        regulation of chemokine production
## 153                                     positive regulation of carbohydrate metabolic process
## 154                                                 fatty acid derivative biosynthetic process
## 155                                                                peptide hormone secretion
## 156                    regulation of systemic arterial blood pressure mediated by a chemical signal
## 157                                         cellular monovalent inorganic cation homeostasis
## 158                                                               cardiac muscle contraction
## 159                                                                vitamin metabolic process
## 160                                                        oligosaccharide metabolic process
## 161                                                           carbohydrate metabolic process
## 162                                                         endomembrane system organization
## 163                                                  cellular carbohydrate metabolic process
## 164                                                          regulation of hormone secretion
## 165                                                                  tight junction assembly
## 166                                                             acyl-CoA biosynthetic process
## 167                                                                   diaphragm contraction
## 168                                  positive regulation of myeloid leukocyte mediated immunity
## 169                                                                 seryl-tRNA aminoacylation
## 170                                                 DNA damage induced protein phosphorylation
## 171                                     positive regulation of actin filament depolymerization
## 172                                       positive regulation of superoxide anion generation
## 173                          establishment or maintenance of epithelial cell apical/basal polarity
## 174                                                                 stem cell fate commitment
## 175                                             negative regulation of dendrite morphogenesis
## 176                                                                 hard palate development
## 177                                           positive regulation of phagocytosis, engulfment
## 178                                                         axonemal dynein complex assembly
## 179                                                         inflammatory response to wounding
## 180                                                        regulation of eosinophil activation
## 181                                                          sulfur compound biosynthetic process
## 182                                                     phosphatidylinositol metabolic process
## 183                                                                regulated secretory pathway
## 184                                                                  hexose metabolic process
## 185                                                    regulation of interleukin-8 production
```

```
## 186                                              photoreceptor cell differentiation
## 187                                                               peptide transport
## 188                                            glycerolipid biosynthetic process
## 189                                                              neuron development
## 190                                         regulation of cellular component size
## 191                                                         multi-organism behavior
## 192                        negative regulation of cellular component organization
## 193  negative regulation of platelet-derived growth factor receptor signaling pathway
## 194                                    peripheral nervous system axon regeneration
## 195                                                         optic nerve development
## 196                                                                     peristalsis
## 197                   positive regulation of toll-like receptor 4 signaling pathway
## 198                                                        bone marrow development
## 199                                 negative regulation of focal adhesion assembly
## 200    epithelial cilium movement involved in determination of left/right asymmetry
## 201                 positive regulation of DNA-templated transcription, initiation
## 202        regulation of N-methyl-D-aspartate selective glutamate receptor activity
## 203                     positive regulation of endothelial cell apoptotic process
## 204                                   cytokine production involved in immune response
## 205                                                               nerve development
## 206                                              regulation of cytokine secretion
## 207                                                            developmental growth
## 208                                             regulation of protein localization
## 209                                                cellular lipid metabolic process
## 210                                                    regulation of system process
## 211                                               triglyceride biosynthetic process
## 212                                                     actin filament-based process
## 213                                                            renal system process
## 214                                           activation of phospholipase C activity
## 215                                     negative regulation of phosphatase activity
## 216                                                 regulation of blood coagulation
## 217                                              neutral lipid biosynthetic process
## 218                                          regulation of phospholipase C activity
## 219                                        regulation of apoptotic signaling pathway
## 220                                                        cell part morphogenesis
## 221                                          regulation of primary metabolic process
## 222                                         positive regulation of heart contraction
## 223                                       regulation of cell projection organization
## 224                                       cellular response to tumor necrosis factor
## 225                                          protein localization to plasma membrane
## 226                                 eosinophil activation involved in immune response
## 227                                                    eosinophil mediated immunity
## 228                                                      arginine catabolic process
## 229                                                 sphingomyelin metabolic process
## 230                                                      membrane raft organization
## 231                                   dermatan sulfate proteoglycan metabolic process
## 232                                    regulation of killing of cells of other organism
## 233                                                 killing by host of symbiont cells
## 234                           negative regulation of protein tyrosine kinase activity
## 235                              establishment or maintenance of bipolar cell polarity
## 236                                              regulation of spindle organization
## 237                                                     regulation of blood pressure
## 238                                                      response to corticosteroid
## 239                                                                      locomotion
```

```
## 240                                                     striated muscle cell differentiation
## 241                                                          oxidation-reduction process
## 242                                                        regulation of ion homeostasis
## 243                                        negative regulation of immune system process
## 244                                                                     hormone transport
## 245                                                                      metabolic process
## 246                                       regulation of actin cytoskeleton organization
## 247                                                            oxoacid metabolic process
## 248                                                          cellular cation homeostasis
```

GO enrichment analysis for **MF**

```
ontology(paraGO) <- "MF"
MF <- hyperGTest(paraGO)
summary(MF)[,c(1,2,7)]
```

```
##        GOMFID       Pvalue
## 1  GO:0000026 6.848844e-07
## 2  GO:0015057 2.721796e-06
## 3  GO:0003727 2.567893e-05
## 4  GO:0004313 7.849775e-05
## 5  GO:0004315 7.849775e-05
## 6  GO:0004316 7.849775e-05
## 7  GO:0004317 7.849775e-05
## 8  GO:0004319 7.849775e-05
## 9  GO:0004320 7.849775e-05
## 10 GO:0016295 7.849775e-05
## 11 GO:0016296 7.849775e-05
## 12 GO:0004314 2.341235e-04
## 13 GO:0016420 2.341235e-04
## 14 GO:0031681 2.341235e-04
## 15 GO:0031894 2.341235e-04
## 16 GO:0001653 2.890821e-04
## 17 GO:0031177 4.655251e-04
## 18 GO:0035064 5.982992e-04
## 19 GO:0000036 7.713683e-04
## 20 GO:0051192 7.713683e-04
## 21 GO:0016417 1.150336e-03
## 22 GO:0005000 2.122460e-03
## 23 GO:0008271 2.122460e-03
## 24 GO:0070402 2.713068e-03
## 25 GO:0017046 2.991566e-03
## 26 GO:0016888 4.097126e-03
## 27 GO:0016887 5.408714e-03
## 28 GO:0001965 5.743499e-03
## 29 GO:0004056 8.905043e-03
## 30 GO:0004462 8.905043e-03
## 31 GO:0004573 8.905043e-03
## 32 GO:0004967 8.905043e-03
## 33 GO:0005200 1.162867e-02
## 34 GO:1901682 1.216228e-02
## 35 GO:0016829 1.518246e-02
## 36 GO:0000340 1.773159e-02
```

```
## 37 GO:0004342 1.773159e-02
## 38 GO:0004487 1.773159e-02
## 39 GO:0004558 1.773159e-02
## 40 GO:0030292 1.773159e-02
## 41 GO:0051424 1.773159e-02
## 42 GO:0097016 1.773159e-02
## 43 GO:0097159 1.855111e-02
## 44 GO:0000166 2.052172e-02
## 45 GO:0033218 2.307389e-02
## 46 GO:0016758 2.528449e-02
## 47 GO:0004930 2.530484e-02
## 48 GO:1901363 2.564289e-02
## 49 GO:0019900 2.573614e-02
## 50 GO:0004477 2.648032e-02
## 51 GO:0004828 2.648032e-02
## 52 GO:0050998 2.648032e-02
## 53 GO:0005080 2.904388e-02
## 54 GO:0016836 2.904388e-02
## 55 GO:0016787 2.924347e-02
## 56 GO:0004767 3.515192e-02
## 57 GO:0050700 3.515192e-02
## 58 GO:0005524 3.547224e-02
## 59 GO:0030554 4.065333e-02
## 60 GO:0016798 4.242920e-02
## 61 GO:0015926 4.331240e-02
## 62 GO:0004396 4.374706e-02
## 63 GO:0004488 4.374706e-02
##                                                                                     Term
## 1                                                alpha-1,2-mannosyltransferase activity
## 2                                                            thrombin receptor activity
## 3                                                             single-stranded RNA binding
## 4                                    [acyl-carrier-protein] S-acetyltransferase activity
## 5                                     3-oxoacyl-[acyl-carrier-protein] synthase activity
## 6                              3-oxoacyl-[acyl-carrier-protein] reductase (NADPH) activity
## 7                           3-hydroxypalmitoyl-[acyl-carrier-protein] dehydratase activity
## 8                      enoyl-[acyl-carrier-protein] reductase (NADPH, B-specific) activity
## 9                                      oleoyl-[acyl-carrier-protein] hydrolase activity
## 10                                   myristoyl-[acyl-carrier-protein] hydrolase activity
## 11                                   palmitoyl-[acyl-carrier-protein] hydrolase activity
## 12                                  [acyl-carrier-protein] S-malonyltransferase activity
## 13                                                         malonyltransferase activity
## 14                                                      G-protein beta-subunit binding
## 15                                                    V1A vasopressin receptor binding
## 16                                                           peptide receptor activity
## 17                                                          phosphopantetheine binding
## 18                                                           methylated histone binding
## 19 ACP phosphopantetheine attachment site binding involved in fatty acid biosynthetic process
## 20                                                            prosthetic group binding
## 21                                                          S-acyltransferase activity
## 22                                                        vasopressin receptor activity
## 23                             secondary active sulfate transmembrane transporter activity
## 24                                                                        NADPH binding
## 25                                                               peptide hormone binding
## 26                          endodeoxyribonuclease activity, producing 5'-phosphomonoesters
```

```
## 27                                                    ATPase activity
## 28                                     G-protein alpha-subunit binding
## 29                                    argininosuccinate lyase activity
## 30                                   lactoylglutathione lyase activity
## 31                      mannosyl-oligosaccharide glucosidase activity
## 32                                         glucagon receptor activity
## 33                               structural constituent of cytoskeleton
## 34                 sulfur compound transmembrane transporter activity
## 35                                                      lyase activity
## 36                                   RNA 7-methylguanosine cap binding
## 37                              glucosamine-6-phosphate deaminase activity
## 38            methylenetetrahydrofolate dehydrogenase (NAD+) activity
## 39                                     alpha-1,4-glucosidase activity
## 40                             protein tyrosine kinase inhibitor activity
## 41                               corticotropin-releasing hormone binding
## 42                                                   L27 domain binding
## 43                                       organic cyclic compound binding
## 44                                                   nucleotide binding
## 45                                                        amide binding
## 46               transferase activity, transferring hexosyl groups
## 47                                    G-protein coupled receptor activity
## 48                                        heterocyclic compound binding
## 49                                                       kinase binding
## 50                   methenyltetrahydrofolate cyclohydrolase activity
## 51                                          serine-tRNA ligase activity
## 52                                         nitric-oxide synthase binding
## 53                                              protein kinase C binding
## 53                                              protein kinase C binding
## 54                                                 hydro-lyase activity
## 55                                                   hydrolase activity
## 56                           sphingomyelin phosphodiesterase activity
## 57                                                  CARD domain binding
## 58                                                          ATP binding
## 59                                            adenyl nucleotide binding
## 60                            hydrolase activity, acting on glycosyl bonds
## 61                                                 glucosidase activity
## 62                                                  hexokinase activity
## 63           methylenetetrahydrofolate dehydrogenase (NADP+) activity
```

GO enrichment analysis for **CC**

```
ontology(paraGO) <- "CC"
CC <- hyperGTest(paraGO)
summary(CC)[,c(1,2,7)]
```

```
##        GOCCID       Pvalue
## 1  GO:0035102 1.662649e-05
## 2  GO:0005783 2.771039e-04
## 3  GO:0005737 6.205058e-04
## 4  GO:0005794 6.358349e-04
## 5  GO:0005622 9.076359e-04
## 6  GO:0042587 1.280729e-03
## 7  GO:0043227 1.724776e-03
## 8  GO:0005623 2.283221e-03
```

```
## 9  GO:0016235 3.285612e-03
## 10 GO:0043229 5.918317e-03
## 11 GO:0005923 6.125909e-03
## 12 GO:0000323 6.612909e-03
## 13 GO:0000133 7.959619e-03
## 14 GO:0005618 7.959619e-03
## 15 GO:0005767 7.959619e-03
## 16 GO:0009941 1.585665e-02
## 17 GO:0034098 1.585665e-02
## 18 GO:0035748 1.585665e-02
## 19 GO:0031300 1.611572e-02
## 20 GO:0000792 1.797360e-02
## 21 GO:0030173 2.069186e-02
## 22 GO:0009506 2.369158e-02
## 23 GO:0009570 2.369158e-02
## 24 GO:0097025 2.369158e-02
## 25 GO:0005902 2.817444e-02
## 26 GO:0098588 2.830548e-02
## 27 GO:0042175 3.074791e-02
## 28 GO:0031045 3.146488e-02
## 29 GO:0043219 3.146488e-02
## 30 GO:0043220 3.146488e-02
## 31 GO:0033268 3.917705e-02
## 32 GO:0035145 3.917705e-02
## 33 GO:0043196 3.917705e-02
## 34 GO:0044435 3.917705e-02
## 35 GO:0005765 4.579720e-02
## 36 GO:0030054 4.679293e-02
## 37 GO:0005829 4.796554e-02
## 38 GO:0005887 4.887769e-02
##                                                             Term
## 1                                                   PRC1 complex
## 2                                          endoplasmic reticulum
## 3                                                      cytoplasm
## 4                                                Golgi apparatus
## 5                                                  intracellular
## 6                                               glycogen granule
## 7                                    membrane-bounded organelle
## 8                                                           cell
## 9                                                     aggresome
## 10                                      intracellular organelle
## 11                                                tight junction
## 12                                                 lytic vacuole
## 13                                                    polarisome
## 14                                                     cell wall
## 15                                           secondary lysosome
## 16                                         chloroplast envelope
## 17                           Cdc48p-Npl4p-Ufd1p AAA ATPase complex
## 18                                  myelin sheath abaxonal region
## 19                      intrinsic component of organelle membrane
## 20                                               heterochromatin
## 21                          integral component of Golgi membrane
## 22                                                   plasmodesma
## 23                                            chloroplast stroma
```

```
## 24                                        MPP7-DLG1-LIN7 complex
## 25                                                    microvillus
## 26                                  bounding membrane of organelle
## 27 nuclear outer membrane-endoplasmic reticulum membrane network
## 28                                               dense core granule
## 29                                                    lateral loop
## 30                                    Schmidt-Lanterman incisure
## 31                                                node of Ranvier
## 32                                    exon-exon junction complex
## 33                                                     varicosity
## 34                                                   plastid part
## 35                                             lysosomal membrane
## 36                                                  cell junction
## 37                                                        cytosol
## 38                           integral component of plasma membrane
```

4. MeSH enrichment analysis

Then, we perform a MeSH ORA for the category **Chemicals and Drugs** by setting 'category="D"'.

```
library(meshr)
library(MeSH.db)
library("org.MeSH.Ssc.db")
meshParams <- new("MeSHHyperGParams", geneIds = my.geneID2[,2], universeGeneIds = univ.geneID3[,2],
                  annotation = "org.MeSH.Ssc.db", category = "D", database = "gene2pubmed",
                  pvalueCutoff = 0.05, pAdjust = "none")
meshR <- meshHyperGTest(meshParams)
summary(meshR)[!duplicated(summary(meshR)[,7]),c(1,2,7)]
```

```
##      MESHID      Pvalue                          MESHTERM
## 1   D000213 0.008353484              Acyl Carrier Protein
## 210 D048630 0.008353484              Polyketide Synthases
## 214 D054890 0.008353484          Fatty Acid Synthase, Type I
## 17  D002245 0.016637643                    Carbon Dioxide
## 169 D019298 0.016637643               Glucose-6-Phosphate
## 211 D051274 0.016637643          Glucose Transporter Type 3
## 101 D006593 0.024853048                        Hexokinase
## 105 D006601 0.024853048                           Hexoses
## 115 D008780 0.024853048                 Methyltransferases
## 159 D018027 0.024853048                Receptors, Glucagon
## 201 D039601 0.024853048    Eukaryotic Initiation Factor-4A
## 233 D064429 0.024853048              Fatty Acid Synthases
## 55  D004763 0.033000266             Glucagon-Like Peptides
## 109 D006629 0.033000266                          Hirudins
## 204 D044463 0.033000266                     Receptor, PAR-1
## 3   D000577 0.041079859                            Amides
## 48  D002457 0.041079859                     Cell Extracts
## 60  D006003 0.041079859                          Glycogen
## 119 D011770 0.041079859                    Pyruvate Kinase
## 143 D012799 0.041079859                 Sialyltransferases
## 153 D016211 0.041079859 Transforming Growth Factor alpha
## 162 D019158 0.041079859           N-Acetylneuraminic Acid
## 172 D019344 0.049092385                       Lactic Acid
```

Switching to a different category is easily done by the 'category<-' function. Here, we use **Diseases** (category = "C").

```
category(meshParams) <- "C"
meshR <- meshHyperGTest(meshParams)
summary(meshR)[!duplicated(summary(meshR)[,7]),c(1,2,7)]
```

MeSH ORA for **Anatomy** (category = "A").

```
category(meshParams) <- "A"
meshR <- meshHyperGTest(meshParams)
summary(meshR)[!duplicated(summary(meshR)[,7]),c(1,2,7)]
```

```
##    MESHID     Pvalue           MESHTERM
## 1 D008841 0.03300027 Actin Cytoskeleton
```

MeSH ORA for **Phenomena and Processes** (category = "G").

```
category(meshParams) <- "G"
meshR <- meshHyperGTest(meshParams)
summary(meshR)[!duplicated(summary(meshR)[,7]),c(1,2,7)]
```

```
##     MESHID     Pvalue         MESHTERM
## 39 D020134 0.02273332 Catalytic Domain
## 1  D004542 0.02485305      Ejaculation
## 24 D013076 0.02485305      Sperm Count
```

5. Session Information

```
sessionInfo()
```

```
## R version 3.1.2 (2014-10-31)
## Platform: x86_64-apple-darwin13.4.0 (64-bit)
##
## locale:
## [1] en_US.UTF-8/en_US.UTF-8/en_US.UTF-8/C/en_US.UTF-8/en_US.UTF-8
##
## attached base packages:
##  [1] grid      parallel  stats4    stats     graphics  grDevices utils
##  [8] datasets  methods   base
##
## other attached packages:
##  [1] org.MeSH.Ssc.db_1.2.0     meshr_1.2.2
##  [3] org.MeSH.Syn.db_1.2.0     org.MeSH.Bsu.168.db_1.2.0
##  [5] org.MeSH.Atu.K84.db_1.2.0 org.MeSH.Aca.db_1.2.0
##  [7] org.MeSH.Hsa.db_1.2.0     MeSH.PCR.db_1.2.0
##  [9] MeSH.AOR.db_1.2.0         MeSH.db_1.2.0
## [11] MeSHDbi_1.2.0             org.Hs.eg.db_3.0.0
## [13] cummeRbund_2.8.2          Gviz_1.10.2
## [15] rtracklayer_1.26.2        GenomicRanges_1.18.3
```

```
## [17] fastcluster_1.1.13        reshape2_1.4
## [19] ggplot2_1.0.0             fdrtool_1.2.13
## [21] GOstats_2.32.0            graph_1.44.0
## [23] Category_2.32.0           GO.db_3.0.0
## [25] Matrix_1.1-4              org.Ss.eg.db_3.0.0
## [27] RSQLite_1.0.0             DBI_0.3.1
## [29] AnnotationDbi_1.28.1      GenomeInfoDb_1.2.3
## [31] IRanges_2.0.0             S4Vectors_0.4.0
## [33] Biobase_2.26.0            BiocGenerics_0.12.1
## [35] biomaRt_2.22.0
##
## loaded via a namespace (and not attached):
##  [1] acepack_1.3-3.3         annotate_1.44.0
##  [3] AnnotationForge_1.8.1   base64enc_0.1-2
##  [5] BatchJobs_1.5           BBmisc_1.8
##  [7] BiocParallel_1.0.0      Biostrings_2.34.0
##  [9] biovizBase_1.14.0       bitops_1.0-6
## [11] brew_1.0-6              BSgenome_1.34.0
## [13] checkmate_1.5.0         cluster_1.15.3
## [15] codetools_0.2-9         colorspace_1.2-4
## [17] dichromat_2.0-0         digest_0.6.4
## [19] evaluate_0.5.5          fail_1.2
## [21] foreach_1.4.2           foreign_0.8-61
## [23] formatR_1.0             Formula_1.1-2
## [25] genefilter_1.48.1       GenomicAlignments_1.2.1
## [27] GenomicFeatures_1.18.2  GSEABase_1.28.0
## [29] gtable_0.1.2            Hmisc_3.14-5
## [31] htmltools_0.2.6         iterators_1.0.7
## [33] knitr_1.8               lattice_0.20-29
## [35] latticeExtra_0.6-26     MASS_7.3-35
## [37] matrixStats_0.10.3      munsell_0.4.2
## [39] nnet_7.3-8              plyr_1.8.1
## [41] proto_0.3-10            R.methodsS3_1.6.1
## [43] RBGL_1.42.0             RColorBrewer_1.0-5
## [45] Rcpp_0.11.3             RCurl_1.95-4.3
## [47] rmarkdown_0.3.10        rpart_4.1-8
## [49] Rsamtools_1.18.2        scales_0.2.4
## [51] sendmailR_1.2-1         splines_3.1.2
## [53] stringr_0.6.2           survival_2.37-7
## [55] tools_3.1.2             VariantAnnotation_1.12.4
## [57] XML_3.98-1.1            xtable_1.7-4
## [59] XVector_0.6.0           yaml_2.1.13
## [61] zlibbioc_1.12.0
```
