# Supplementary material for: An application of MeSH enrichment analysis in livestock
Source: Anim Genet. 2015 Jun 2;46(4):381–7. doi: 10.1111/age.12307 (PMC5032990; doi:10.1111/age.12307)
Supplement: Supplementary file 3 — Appendix S3 MeSH over‐representation analysis (Horse) [file AGE-46-381-s003.pdf]

APPENDIX S3

MeSH over-representation analysis (Horse)

This is a reproducible report written by an R Markdown.

1. Obtain a list of genes

```
dat <- read.table("quarter.txt", header=FALSE, stringsAsFactors=FALSE)

require(biomaRt)
mart <- useMart(biomart = "ensembl", dataset = "ecaballus_gene_ensembl")
## gene annotation
allgenes <-getBM(c("ensembl_gene_id", "entrezgene", "hgnc_id", "start_position", "end_position",
                   "chromosome_name"), mart = mart)

chr.name<-numeric()
bp.loc <- numeric()
ensembl.id<-numeric()
entrez.id<-numeric()

m<-1
for(k in 1:nrow(dat))
{
  sub.gene <- subset(allgenes, chromosome_name == dat[k,1])
    for(j in 1:nrow(sub.gene))
    {
            if((sub.gene$start_position[j] - 250000) <= (dat[k,2]) &
           (sub.gene$end_position[j] + 250000) >= (dat[k,2]))
            {
                chr.name[m]<-dat[k,1]
                bp.loc[m]<-dat[k,2]
                ensembl.id[m]<-sub.gene$ensembl_gene_id[j]
                entrez.id[m]<-sub.gene$entrezgene[j]
                m<-m+1
            }
        }
    }
```

2. Create a vector of background (universe) genes

We first create a vector of all genes.

```
## remove genes with no corresponding Entrez Gene ID
allgenes2 <- allgenes[!is.na(allgenes$entrezgene),] # 17175
## remove duplicated Entrez Gene ID
allgenes3 <- allgenes2[ !duplicated(allgenes2$entrezgene),] # 16808
```

3. Create a vector of significant genes

Secondly, we create a vector of significant genes.

```
dat2 <- data.frame(chr.name, bp.loc, ensembl.id, entrez.id) # 179
## remove genes with no corresponding Entrez Gene ID
dat3 <- dat2[!is.na(dat2$entrez.id),] # 113
```

4. MeSH enrichment analysis

Then, we perform a MeSH ORA for the category **Chemicals and Drugs** by setting 'category="D"'.

```
library(meshr)
library(MeSH.db)
library("org.MeSH.Eqc.db")
meshParams <- new("MeSHHyperGParams", geneIds = as.integer(dat3$entrez.id),
                  universeGeneIds = allgenes3$entrezgene, annotation = "org.MeSH.Eqc.db",
                  category = "D", database = "gene2pubmed", pvalueCutoff = 0.05, pAdjust = "none")
meshR <- meshHyperGTest(meshParams)
summary(meshR)[!duplicated(summary(meshR)[,7]),c(1,2,7)]
```

```
##     MESHID      Pvalue                                 MESHTERM
## 68 D017392 0.006722989   Thiobarbituric Acid Reactive Substances
## 79 D055435 0.006722989                                 Myostatin
## 1  D000521 0.013401177                                 alpha-MSH
## 69 D017471 0.013401177                     Receptors, Interferon
## 71 D018389 0.013401177                           Codon, Nonsense
## 73 D021901 0.013401177                           DNA, Intergenic
## 75 D047888 0.013401177  Receptors, Tumor Necrosis Factor, Type I
## 77 D047889 0.013401177 Receptors, Tumor Necrosis Factor, Type II
## 13 D009842 0.033169882                             Oligopeptides
## 18 D015232 0.039671804                              Dinoprostone
## 24 D015237 0.039671804                                 Dinoprost
## 30 D015395 0.039671804       Histocompatibility Antigens Class I
## 44 D015850 0.039671804                             Interleukin-6
## 3  D007375 0.046130386                             Interleukin-1
## 55 D016753 0.046130386                            Interleukin-10
```

Switching to a different category is easily done by the 'category<-' function. Here, we use **Diseases** (category = "C").

```
category(meshParams) <- "C"
meshR <- meshHyperGTest(meshParams)
summary(meshR)[!duplicated(summary(meshR)[,7]),c(1,2,7)]
```

```
##    MESHID     Pvalue                  MESHTERM
## 1 D008173 0.02003486 Lung Diseases, Obstructive
```

MeSH ORA for **Anatomy** (category = "A").

```
category(meshParams) <- "A"
meshR <- meshHyperGTest(meshParams)
summary(meshR)[!duplicated(summary(meshR)[,7]),c(1,2,7)]
```

```
##     MESHID      Pvalue         MESHTERM
## 15 D009434 0.006722989  Neural Pathways
## 16 D013116 0.006722989      Spinal Cord
## 12 D008184 0.020034859     Luteal Cells
## 1  D004847 0.046130386 Epithelial Cells
```

MeSH ORA for **Phenomena and Processes** (category = "G").

```
category(meshParams) <- "G"
meshR <- meshHyperGTest(meshParams)
summary(meshR)[!duplicated(summary(meshR)[,7]),c(1,2,7)]
```

```
##      MESHID      Pvalue                        MESHTERM
## 79  D006239 0.005704067                      Haplotypes
## 118 D010805 0.006389037    Physical Conditioning, Animal
## 57  D005684 0.006722989                            Gait
## 117 D009043 0.006722989                  Motor Activity
## 150 D011597 0.006722989        Psychomotor Performance
## 153 D013008 0.006722989                    Somatotypes
## 163 D020029 0.006722989                   Base Pairing
## 151 D012420 0.013401177                        Running
## 157 D018389 0.013401177                Codon, Nonsense
## 159 D019898 0.013401177        Autocrine Communication
## 161 D019899 0.013401177        Paracrine Communication
## 164 D021901 0.013401177               DNA, Intergenic
## 154 D015810 0.020034859         Linkage Disequilibrium
## 1   D003341 0.026624330                    Luteolysis
## 58  D005784 0.026624330             Gene Amplification
## 146 D010807 0.026624330             Physical Endurance
## 5   D005091 0.030980920                          Exons
## 110 D008285 0.033169882 Major Histocompatibility Complex
## 102 D006579 0.039671804                   Heterozygote
## 63  D005805 0.046130386             Genes, MHC Class I
```

5. Session Information

```
sessionInfo()
```

```
## R version 3.1.2 (2014-10-31)
## Platform: x86_64-apple-darwin13.4.0 (64-bit)
##
## locale:
## [1] en_US.UTF-8/en_US.UTF-8/en_US.UTF-8/C/en_US.UTF-8/en_US.UTF-8
##
## attached base packages:
##  [1] grid      parallel  stats4    stats     graphics  grDevices utils
##  [8] datasets  methods   base
##
## other attached packages:
##  [1] org.MeSH.Eqc.db_1.2.0     meshr_1.2.2
##  [3] org.MeSH.Syn.db_1.2.0     org.MeSH.Bsu.168.db_1.2.0
```

```
##  [5] org.MeSH.Atu.K84.db_1.2.0 org.MeSH.Aca.db_1.2.0
##  [7] org.MeSH.Hsa.db_1.2.0     MeSH.PCR.db_1.2.0
##  [9] MeSH.AOR.db_1.2.0         MeSH.db_1.2.0
## [11] MeSHDbi_1.2.0             org.Hs.eg.db_3.0.0
## [13] cummeRbund_2.8.2          Gviz_1.10.2
## [15] rtracklayer_1.26.2        GenomicRanges_1.18.3
## [17] fastcluster_1.1.13        reshape2_1.4
## [19] ggplot2_1.0.0             Category_2.32.0
## [21] GO.db_3.0.0               RSQLite_1.0.0
## [23] DBI_0.3.1                 AnnotationDbi_1.28.1
## [25] GenomeInfoDb_1.2.3        IRanges_2.0.0
## [27] S4Vectors_0.4.0           Biobase_2.26.0
## [29] BiocGenerics_0.12.1       Matrix_1.1-4
## [31] fdrtool_1.2.13            biomaRt_2.22.0
##
## loaded via a namespace (and not attached):
##  [1] acepack_1.3-3.3          annotate_1.44.0
##  [3] base64enc_0.1-2          BatchJobs_1.5
##  [5] BBmisc_1.8               BiocParallel_1.0.0
##  [7] Biostrings_2.34.0        biovizBase_1.14.0
##  [9] bitops_1.0-6             brew_1.0-6
## [11] BSgenome_1.34.0          checkmate_1.5.0
## [13] cluster_1.15.3           codetools_0.2-9
## [15] colorspace_1.2-4         dichromat_2.0-0
## [17] digest_0.6.4             evaluate_0.5.5
## [19] fail_1.2                 foreach_1.4.2
## [21] foreign_0.8-61           formatR_1.0
## [23] Formula_1.1-2            genefilter_1.48.1
## [25] GenomicAlignments_1.2.1  GenomicFeatures_1.18.2
## [27] graph_1.44.0             GSEABase_1.28.0
## [29] gtable_0.1.2             Hmisc_3.14-5
## [31] htmltools_0.2.6          iterators_1.0.7
## [33] knitr_1.8                lattice_0.20-29
## [35] latticeExtra_0.6-26      MASS_7.3-35
## [37] matrixStats_0.10.3       munsell_0.4.2
## [39] nnet_7.3-8               plyr_1.8.1
## [41] proto_0.3-10             R.methodsS3_1.6.1
## [43] RBGL_1.42.0              RColorBrewer_1.0-5
## [45] Rcpp_0.11.3              RCurl_1.95-4.3
## [47] rmarkdown_0.3.10         rpart_4.1-8
## [49] Rsamtools_1.18.2         scales_0.2.4
## [51] sendmailR_1.2-1          splines_3.1.2
## [53] stringr_0.6.2            survival_2.37-7
## [55] tools_3.1.2              VariantAnnotation_1.12.4
## [57] XML_3.98-1.1             xtable_1.7-4
## [59] XVector_0.6.0            yaml_2.1.13
## [61] zlibbioc_1.12.0
```
